# Molecular Engineering of Aptamers for Glioblastoma Therapy: From Simple Antagonists to AI-Driven Approaches, a Narrative Review

**DOI:** 10.3390/ijms27188231

**Published:** 2026-09-16

**Authors:** Luana Di Leandro, Martina Colasante, Mariano Catanesi, Francesco Giansanti, Annamaria Cimini, Michele D’Angelo, Vanessa Castelli, Rocco Savino, Rodolfo Ippoliti

**Affiliations:** 1 Department of Life, Health and Environmental Sciences, University of L’Aquila, 67100 L’Aquila, Italy; mcolasante@unite.it (M.C.); mariano.catanesi@univaq.it (M.C.); francesco.giansanti@univaq.it (F.G.); annamaria.cimini@univaq.it (A.C.); michele.dangelo@univaq.it (M.D.); vanessa.castelli@univaq.it (V.C.); 2 Sbarro Institute for Cancer Research and Molecular Medicine, Department of Biology, Temple University, Philadelphia, PA 19122, USA; 3 Department of Medical and Surgical Sciences, Magna Graecia University, 88100 Catanzaro, Italy; savino@unicz.it

**Keywords:** glioblastoma, aptamers, targeted drug delivery, nanostructures, generative machine learning

## Abstract

Glioblastoma multiforme (GBM) is an extremely aggressive and lethal brain tumor, characterized by marked molecular heterogeneity, the persistence of glioma stem cells (GSCs), and the limited permeability of the blood–brain barrier (BBB), which collectively hinder therapeutic efficacy. To address these barriers, nucleic acid aptamers, short single-stranded oligonucleotides with high affinity and specificity for molecular targets, have emerged as a promising therapeutic platform. Early unmodified aptamers, such as AS1411 and U2, demonstrated target engagement but showed limited performance due to instability and rapid systemic clearance. Chemical modifications, including 2′-fluoro substitutions and PEGylation, resulted in improved stability, specificity, and pharmacokinetic properties, enabling the development of innovative aptamer drug conjugates (ApDCs) for targeted delivery to GBM cells. In parallel, multivalent aptamer architectures, such as bispecific aptamer targeting entities (BATEs) and aptamer guided nanostructures, have been designed to enhance binding avidity, address tumor heterogeneity, and facilitate BBB transcytosis. More recently, computational strategies ranging from machine learning-guided sequence optimization to structure prediction and generative AI have accelerated the rational design of aptamers tailored to GBM specific challenges. This review examines these advances, the remaining pharmacological limitations, and the potential of computational tools to reshape the future of aptamer based GBM therapeutics.

## 1. Introduction

GBM remains the most prevalent and devastating primary malignant brain tumor in adults. Despite the adoption of aggressive multimodal treatment, including surgical resection, radiotherapy, and adjuvant chemotherapy with Temozolomide (TMZ), the prognosis remains dismal, with a median survival of approximately 15 months [1,2,3,4]. The unfavorable clinical outcome and prognosis are due to multiple intrinsic biological and anatomical barriers, including the pronounced molecular heterogeneity of tumor cells, the persistence of therapy-resistant glioblastoma stem cells (GSCs), the immunosuppressive tumor microenvironment, and the obstacle to drug delivery imposed by the blood–brain barrier (BBB) [5,6]. Biologically, GBM tumors are characterized by profound cellular heterogeneity and rapid necrotic expansion resulting from uncontrolled tumor proliferation [7,8]. The presence of GSCs is particularly critical to tumor recurrence and intrinsic resistance to conventional therapies, underscoring the need for targeted agents capable of selectively eliminating this malignant cell population [9]. Moreover, tumor cells actively remodel the surrounding Tumor Microenvironment (TME), upregulating the expression of extracellular matrix proteins (such as Tenascin-C) and growth-promoting factors (such as Platelet-Derived Growth Factor Receptor beta (PDGFRβ), thereby establishing a protective niche that facilitates tumor invasion and progression [10,11].

Aptamers are short, single-stranded DNA or RNA oligonucleotides that can fold into three-dimensional conformations, enabling them to bind target molecules with remarkable selectivity and affinity [12,13]. Their ability to interact with a broad spectrum of biomolecules including proteins, peptides, small molecules, and even entire cells, together with methods of selection have been pioneering described by Ellington and Szostak, in a landmark paper [12]. Aptamer binding interactions are governed by shape complementarity, electrostatic forces, and hydrogen bonding, resulting in dissociation constants within the low nanomolar to picomolar range [14]. From a biophysical perspective, aptamers exhibit substantial advantages over conventional monoclonal antibodies, as they can be chemically synthesized, typically elicit low intrinsic immunogenicity depending on their scaffold and chemical modifications, and demonstrate superior thermal stability relative to antibodies [15]. These properties suggest that aptamers can be considered suitable for applications in targeted therapy, drug delivery, and molecular imaging, even within the intricate pathological context of GBM, characterized by the obstacle of BBB penetration, pronounced tumor heterogeneity, and immune evasion [16]. Aptamer-based therapies exhibit remarkable adaptability in selectively targeting tumor cells and can nowadays be considered as agents of precision medicine. Within this framework, aptamer engineering emerges as a promising strategy to overcome the limitations of conventional cancer therapeutics by enabling the development of engineered, highly selective, and modular platforms that integrate tumor-associated biological features with advanced design principles [16,17,18,19,20].

Two major pharmacological barriers underlie the translational failure of numerous promising GBM therapies: the first is the BBB, an exceptionally selective endothelial interface that excludes more than 98% of small-molecule drugs and virtually all large-molecule biologics from entering the central nervous system, thereby preventing therapeutic agents from attaining effective concentrations within the tumor core [21,22]. In addition, the brain parenchyma is characterized by a blood–tumor barrier (BTB), a crucial pathological change in the glioblastoma microenvironment which, although partially compromised, exhibits marked heterogeneity in permeability [23,24,25]. The BTB is characterized by irregular vessel production, structural remodeling, and the loss of tight junctions, in contrast to the restrictive integrity of a healthy blood–brain barrier (BBB). This disruption, however, is heterogeneous; some areas become extremely accessible, while others maintain a barrier that resembles the typical BBB’s protection function. A ‘patchwork’ of drug accessibility is produced by this spatial variability, which is sustained by regional differences in astrocyte endfeet, pericyte coverage, and vascular density. In the end, this uneven permeability continues to be a significant obstacle to systemic treatments.

The second barrier reflects the intrinsic pharmacokinetic constraints of nucleic acid-based therapeutics. Aptamers, designed as single-stranded oligonucleotides, offer advantages such as small molecular size and relatively simple chemical synthesis; however, their pronounced susceptibility to nuclease-mediated degradation and rapid renal clearance confer short systemic half-lives, substantially limiting the duration of target engagement required to achieve sustained therapeutic efficacy o [26,27].

The convergence of these critical challenges, the need for GSC-specific targeting, the requirement for BBB transcytosis, and the need for stable pharmacokinetics, provides a compelling rationale for innovative product development. Aptamers represent an attractive therapeutic modality owing to their distinctive properties, including high affinity and specificity, minimal immunogenicity, and chemical versatility [19,20,28,29,30]. However, the initial application of unconjugated aptamers as simple molecular antagonists in early studies, which employed oligonucleotides as ‘molecular toxins’ optimized for stability, failed to overcome pharmacokinetic limitations and did not achieve sufficient exposure to confer meaningful efficacy [27]. In this paper, conceived as a narrative review, the primary objective is to provide a conceptual and mechanistic overview of the evolution of aptamer engineering for glioblastoma, from unconjugated antagonists and chemically stabilized aptamers to aptamer–drug conjugates, multivalent nanostructures, and bispecific constructs, with a view towards the future of computationally assisted design.

## 2. Chemical Modifications to Enhance Aptamer Stability and Pharmacology

### 2.1. Key Affinity and Specificity Features

A diverse array of GBM-targeting aptamers supports a multifaceted therapeutic paradigm that integrates molecular antagonism with targeted delivery. Therapeutic targets have been selected based on cell-surface proteins implicated in invasion, proliferation, and critical resistance to standard of care (SoC) therapies. In this context, high-affinity binding is a prerequisite for in vivo efficacy: many candidates display dissociation constants in the low-nanomolar range, enabling robust target engagement in the complex tumor microenvironment. As summarized below (Table 1), prominent aptamers against glioblastoma span targets such as Ephrin type-A receptor 2 (EphA2), Platelet-Derived Growth Factor Receptor (PDGFR), and Epidermal Growth Factor Receptor variant III (EGFRvIII), and operate either as unconjugated antagonists or as targeting ligands that enable precise payload delivery.

The dissociation constant (KD) is a central metric for aptamer utility in therapy, capturing the thermodynamic strength of the binding to the target. Lower KD values reflect higher binding affinity, typically in the picomolar to low-nanomolar range and generally correlate with superior pharmacodynamic performance. However, high affinity does not automatically imply high selectivity or immediate clinical efficacy, as physiological barriers and tumor heterogeneities often heavily influence translational outcomes; while KD measures how tightly an aptamer binds, selectivity describes its ability to discriminate against the target from structurally related proteins or wild-type variants (e.g., EGFRvIII vs. EGFRwt). Therefore, achieving therapeutic precision in GBM requires both sub-nanomolar affinity and rigorous counter-selection strategies to ensure target specificity. As a result, these parameters guide decisions on scaffold refinement, multivalency, and conjugation strategies to meet the demands of GBM’s complex biology and microenvironment majority (Table 1). Notwithstanding the favorable affinity for their biological targets, the vast majority of aptamers reported in Table 1 are under evaluation in preclinical studies and still in vitro testing for GBM treatment, being clinical trials limited to the case of NOX-A12. For clinical-stage candidates, these pre-clinical affinity metrics must be formally validated in human trials, where translational success is ultimately defined by strictly regulated eligibility criteria, cohort sizing, predefined primary endpoints, and controlled survival analyses.

### 2.2. Aptamers as Unconjugated (Monovalent) Antagonists

Therapy with unconjugated, monovalent aptamers is attractive when single-stranded oligonucleotides can serve as molecular antagonists or signaling modulators without additional conjugation. These agents rely on highly specific, conformation-driven interactions with overexpressed cell-surface receptors that support GBM progression and stemness, examples including PDGFR and EphA2. Upon binding, aptamers may sterically hinder ligand association, promote receptor internalization, or indirectly suppress downstream signaling pathways such as PI3K/AKT and Ras/MAPK, thereby restraining proliferation, migration, and survival in the hostile tumor microenvironment [8,11,70].

A paradigmatic example is CL4, which binds the extracellular domain of EGFRvIII, a constitutively active deletion mutant lacking portions of the ligand-binding site [34]. By occupying a critical epitope near the dimerization interface, CL4 sterically hinders EGFRvIII self-association and autophosphorylation at key tyrosine residues, damping PI3K/AKT and MAPK/ERK signaling that drives glioblastoma proliferation and invasion [34]. Similarly, Gint4.T engages PDGFRβ with high affinity and acts as a competitive antagonist to PDGF-BB-mediated ligand engagement and receptor dimerization. This leads to reduced receptor phosphorylation and inhibition of STAT3, AKT, and ERK1/2 activation, ultimately controlling and slowing tumor cell motility, vascular mimicry, and growth in pre-clinical in vitro and ectopic/orthotopic xenograft models [34,35,71].

The AS1411 aptamer, which targets nucleolin, an RNA/DNA-binding protein overexpressed in malignant cells, exerts its effects after internalization by disrupting nucleolin’s regulatory roles in mRNA stabilization and DNA replication [31,72,73,74,75,76,77]. Functionally, this induces cell-cycle arrest, accumulation of reactive oxygen, and apoptosis. Notably, AS1411’s guanine-rich G-quadruplex (G4) confers intrinsic nuclease resistance and unexpected serum stability, enabling selective accumulation in nucleolin-overexpressing tumor cells, including GBM [78,79,80]. Beyond pure antagonism, AS1411’s efficient endocytic internalization suggested its use as an internalizing delivery vehicle for therapeutic payloads such as siRNAs or cytotoxic drugs [79]. However, while AS1411 evaluated safety and significant activity in Phase I and II trials for renal cell carcinoma and acute myeloid leukemia (NCT00740441, NCT00512083), its application in GBM remains strictly restricted to pre-clinical investigation, as no formal clinical trials with defined eligibility criteria or survival endpoints have been conducted for glioma patients. Additional antagonistic aptamers have been identified across a spectrum of signaling and adhesion molecules relevant to aggressive GBM phenotypes all currently operating at the preclinical stage. The U2 aptamer binds to EGFRvIII, inhibiting downstream receptor signaling, reducing cell migration and invasion, promoting apoptosis, and acts as a radiosensitizer within the GBM microenvironment [32]. The A40s RNA aptamer targets the EphA2 signaling axis by directly binding EphA2 on glioblastoma stem cells (GSCs), thereby mitigating receptor autophosphorylation, cell proliferation, and stemness features in pre-clinical GBM models [36,37,38]. GL21.T, initially characterized as an antagonist of AXL receptor tyrosine kinase, suppresses downstream PI3K/AKT signaling and AXL activation, diminishing invasiveness, tumorigenicity, and migration; it also functions as a targeting moiety in conjugate and RNA-interference platforms [49,71]. Finally, GL43.T binds EphB2/B3, receptors overexpressed in GBM and implicated in tumorigenesis and invasion; its rapid internalization by GBM cells is associated with reduced proliferative and migratory capacities, in vitro, albeit with reported KD values in the high nanomolar range for EphB3 and pending deeper characterization for EphB2 [39].

### 2.3. Chemical Stabilization and Functionalization Strategies of Aptamers

#### 2.3.1. Sugar and Backbone Modifications

Unmodified oligonucleotide aptamers are susceptible to rapid degradation by serum nucleases and clearance via renal filtration, necessitating chemical optimization for in vivo use. Substitutions at the ribose 2′ position, 2′-fluoro (2′-F), 2′-O-methyl (2′-OMe), and 2′-amino (2′-NH_2_), are among the most effective strategies to enhance nuclease resistance and thermal stability while preserving target affinity and specificity [81,82,83,84]. For example, Gint4.T incorporates 2′-F–pyrimidines and a 3′-inverted deoxythymidine cap, conferring robust resistance to both exonucleases and endonucleases while maintaining picomolar-range binding. This chemistry underpins potent PDGFRβ pathway inhibition in preclinical models and suppresses tumor growth and vascularization in glioma xenografts. Likewise, GL21.T features 2′-F–pyrimidine modifications that enhance serum stability and extend biological half-life, supporting its dual function as both antagonist and delivery scaffold. GL43.T is a 2′-fluoro pyrimidine-modified RNA aptamer that binds to EphB2 and EphB3 receptors, which are overexpressed in GBM cells and are involved in tumorigenesis and invasion. In its unconjugated form, GL43.T undergoes rapid internalization in GBM cells, thereby reducing proliferative and migratory capacities in vitro. The KD of GL43.T has previously been reported for the EphB3 receptor to be in the high nanomolar range (433.5 nM) suggesting intermediate affinity but requires further detailed investigation to characterize the binding to EphB2 and other Eph family [39].

Beyond sugar modifications, phosphorothioate inter-nucleoside linkages replace a non-bridging oxygen in the phosphate backbone with sulfur, substantially improving resistance to nucleolytic cleavage and sometimes increasing cellular uptake [84,85]. Locked nucleic acids (LNAs), characterized by a methylene bridge between the 2′-oxygen and 4′-carbon, stabilize the sugar pucker, increase duplex rigidity, and enhance both affinity and thermal stability [84,86]. In the case of TTA1 [48,87], LNA incorporation improved plasma stability, stem-loop thermal robustness, tumor uptake, and blood retention in animal biodistribution studies while preserving binding affinity; in contrast, unmodified TTA1 was rapidly cleared via fecal excretion. Collectively, these backbone and sugar chemistries are foundational for candidate stability prior to translational evaluation.

#### 2.3.2. PEGylation and Hydrodynamic Tuning

PEGylation is widely used to increase the hydrodynamic radius of aptamers, thereby reducing glomerular filtration and prolonging systemic circulation [27,84,88]. This approach can markedly improve pharmacokinetic profiles without compromising target recognition. PEGylation increases the molecular weight of aptamers, significantly reducing their renal clearance rate and improving circulation half-life. The high-molecular-weight PEG chains establish a hydrated, sterically crowded physical barrier that effectively blocks nuclease access [84]. PEGylation is also integral to the clinical rationale of NOX-A12 (Olaptesed pegol), where a 40 kDa PEG polymer synergizes with Spiegelmer chemistry to prolong biological half-life and sustain CXCL12/SDF-1 neutralization in vivo [52].

#### 2.3.3. Chemical Conjugation

Transcytosis across BBB remains a central challenge for aptamer therapeutics in GBM. To address limited brain penetration, multiple chemical conjugation strategies have been developed at the pre-clinical level [89,90]. Ligand-mediated transport leverages conjugation to molecules recognized by endothelial receptors that facilitate receptor-mediated transcytosis (RMT) [91]. Aptamer conjugates to transferrin, lactoferrin, or ApoE-mimetic peptides exploit receptors highly expressed on brain capillary endothelium, enabling selective transport into tumor parenchyma in experimental models [58]. Lipid conjugation, including cholesterol, tocopherol, or palmitic acid, increases lipophilicity, improves membrane interactions, and extends circulation time, which collectively enhances the likelihood of BBB crossing. In parallel, covalent attachment of cell-penetrating peptides (CPPs) such as TAT or Angiopep-2 can drive brain targeting via adsorptive-mediated endocytosis [92,93]. Ongoing pre-clinical work increasingly adopts multifunctional constructs that combine PEGylation, receptor-targeting ligands, and nuclease-resistant chemistries to deliver favorable pharmacokinetic and biodistribution profiles without eroding affinity.

#### 2.3.4. Spiegelmer Technology (L-Nucleic Acid Aptamers)

The Spiegelmer approach exemplifies a high-impact stabilization strategy for therapeutic aptamers [27]. Substituting natural D-nucleotides with their L-stereoisomer counterparts inverts chirality, rendering aptamers highly resistant to enzymatic degradation by tissue and blood-borne nucleases and substantially extending in vivo half-life. Spiegelmer preserves high affinity and specificity for their targets while often permitting reduced dosing frequency, an advantage in GBM, where stability and microenvironmental delivery are constrained. Spiegelmer generation typically proceeds by isolating a natural D-aptamer, synthesizing the mirror-image L-enantiomer, and layering additional chemical modifications to further optimize affinity and stability.

Although many GBM-specific aptamer programs remain in preclinical development, their strong target affinities and mechanistic relevance to tumor progression and glioma stem cell (GSC) survival underscore substantial translational potential. Among GBM directed aptamers, NOX-A12 (Olaptesed pegol) is one of the few evaluated in an early phase clinical study. NOX-A12 is a PEGylated L-RNA Spiegelmer that targets the chemokine CXCL12, which orchestrates angiogenesis, immune evasion and drug resistance. By binding and neutralizing CXCL12, NOX-A12 disrupts these axes and can sensitize tumors to modalities such as radiotherapy. The Phase ½ GLORIA trial (NCT04121455) evaluated NOX-A12 in newly diagnosed patients with incompletely resected, MGMT-unmethylated GBM. In a six-patient expansion cohort receiving a combination of radiotherapy, bevacizumab and NOX-A12, a median overall survival of 19.9 months was reported. This figure, derived from a post-hoc comparative analysis in a study whose primary endpoint was safety, represents an encouraging signal; however, it should be noted that this result stems from an uncontrolled cross-trial comparison against historical populations that are not directly comparable. NOX-A12 has received Orphan Drug designation from both FDA and EMA, as well as Fast Track status from the FDA, reflecting regulatory recognition from the unmet medical need and facilitating clinical development [51,52].

#### 2.3.5. Comparative Evaluation of Chemical Optimization Strategies vs. Biologics

For a thorough assessment of aptamer development technologies in relation to traditional therapeutic interventions, Table 2 highlights some of the critical pharmacological and logistical factors among 2′-F, LNA, PEGylation, and Spiegelmer chemistries in comparison to mAbs and CAR-T treatments. Unmodified aptamers have issues of fast renal excretion and degradation by enzymes; however, chemical modification significantly increases the half-life and enzymatic stability of aptamers. Moreover, it is important to note that Spiegelmers are totally resistant to nucleases and are virtually non-immunogenic due to their chiral nature and, thus, help overcome two critical drawbacks of protein-based biotherapies. Also, despite biological persistence of mAbs and cell-based therapies (for example, CAR-T), their practical use is limited by risks of high immunogenicity (formation of anti-drug antibodies or cytokine release syndrome) and expensive manufacturing process.

### 2.4. Criticisms and Perspectives

Despite chemical modification of aptamers has gained substantial progress in their resistance to degradation, the improved biochemical stability should not be considered automatically as synonymous with improved therapeutic performance.

Moreover, the available evidence remains predominantly evaluated on in vitro models or preclinical conditions in non-humans, determining a clear difficulty in the comparison of different stabilization strategies due to heterogeneity in experimental conditions, pharmacokinetic analyses, and GBM models that vary substantially across studies.

Therefore, future studies should evaluate chemical optimization not only in terms of stability and affinity, but through integrated pharmacokinetic, biodistribution, toxicity, BBB-penetration, and therapeutic-efficacy analyses using clinically relevant GBM models.

## 3. From Monovalent Antagonists to Multivalent Platforms: Aptamer-SoC (Standard-of-Care) Synergy

### 3.1. Advanced Aptamer Based Platforms

Chemical modifications have improved aptamer stability and bioavailability, yet single-chain aptamers still show limited durability when used alone against GBM’s complex cellular environment. This has driven a shift toward more sophisticated formulation such as aptamer conjugates (ApCs), in which aptamers retain their targeting function while serving as platforms for the binding of therapeutic payloads. These constructs can carry chemotherapy agents such as doxorubicin or temozolomide, RNA-based silencers, radioactive isotopes, or immunomodulatory molecules, and can also be arranged on nanostructures to enhance cellular uptake [103]. ApCs are designed to interact synergistically with standard-of-care (SoC) treatments chemotherapy and radiotherapy, by improving drug delivery, increasing tumor radiosensitivity, or ensuring co-localization of aptamer payloads and SoC agents. Their modular architecture allows adaptation to different clinical needs while preserving aptamer specificity. Overall, ApCs represent an evolution beyond first generation aptamers, functioning as integrated nanoscale platforms that combine targeting precision, drug delivery, and therapeutic synergy, with the potential to enhance the efficacy of conventional GBM therapies [17,19,104,105].

Multiple strategies now arrange several aptamer units on a single scaffold to enhance avidity, specificity, and functional versatility beyond monovalent formats. These scaffolds may be nucleic-acid–based (DNA or RNA origami) or built from non-nucleic materials such as nanoparticles, dendrimers, polymers, or protein architectures. Presenting multiple ligands in defined spatial configurations increases local target concentration, enables simultaneous receptor engagement, and can generate cooperative effects suited to complex or clustered receptor systems.

Nano-scaffold design also allows precise control of aptamer orientation, density, and spacing. DNA origami and RNA tiles offer programmable nanometer-scale positioning, while dendrimers, polymers, liposomes, and inorganic nanoparticles support high-density conjugation and can co-deliver radiosensitizers, siRNAs, or chemotherapeutics. Protein-based scaffolds, including streptavidin tetramers or engineered antibody fragments, provide defined valency and orientation, with rigidity and linker flexibility tuned to preserve aptamer folding and dynamic receptor engagement.

**Figure 1:**
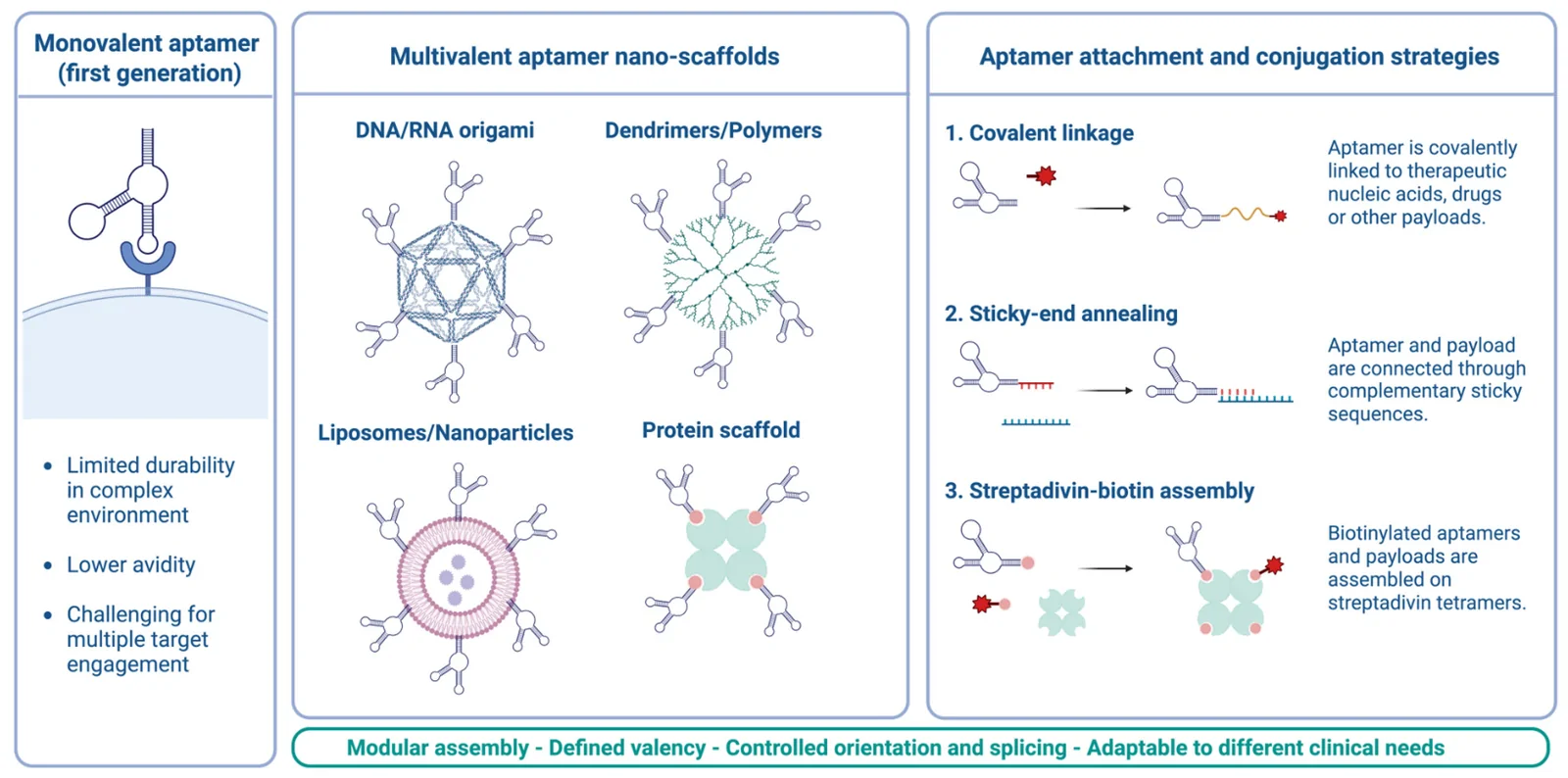
Advanced aptamer-based platforms, highlighting the transition from monovalent aptamers to multivalent nano-scaffolds and their integration with therapeutic cargos through three different conjugation strategies: covalent linkage, sticky-end annealing, streptavidin–biotin assembly. Created in BioRender. Colasante, M. (2026) https://BioRender.com/t3rehd9 (accessed on 14 September 2026).

Together, these nano-scaffold systems convert aptamers into polyvalent nanoscale constructs capable of integrating targeting, drug or siRNA delivery, radiosensitizer transport, and adjuvant functions, helping compensate for the limitations of monovalent aptamers in the GBM microenvironment (Table 3, Figure 1).

The following subsections examine how aptamer architectures such as Nucleic Acid Therapeutic (NAT)-aptamer conjugates and nano-scaffolds evolved, enhancing the cellular response to the GBM SoC. We focus on four key areas: the development of NAT-based conjugates followed by strategies to sensitize tumor cells to chemotherapy, radiotherapy, and Tumor Treating Fields (TTFields). Together, these approaches show how molecular precision can be leveraged to overcome therapeutic resistance.

### 3.2. Aptamer-NAT Conjugates

Nucleic acid therapies (NATs) have become important tools in precision oncology, enabling targeted modulation of cancer-associated gene expression, and several NAT-based strategies have already entered clinical investigation for glioblastoma (GBM) [106,107,108,109,110].

These modalities include plasmid DNA, siRNAs, anti-miRNAs, miRNA mimics, mRNA, catalytic nucleic acids, and CRISPR–Cas9 guide RNAs, each acting through distinct mechanisms. NATs such as siRNAs allow selective inhibition of oncogenic pathways that are inaccessible to small-molecule drugs, reducing toxicity to healthy tissues through highly specific target engagement.

**Figure 2:**
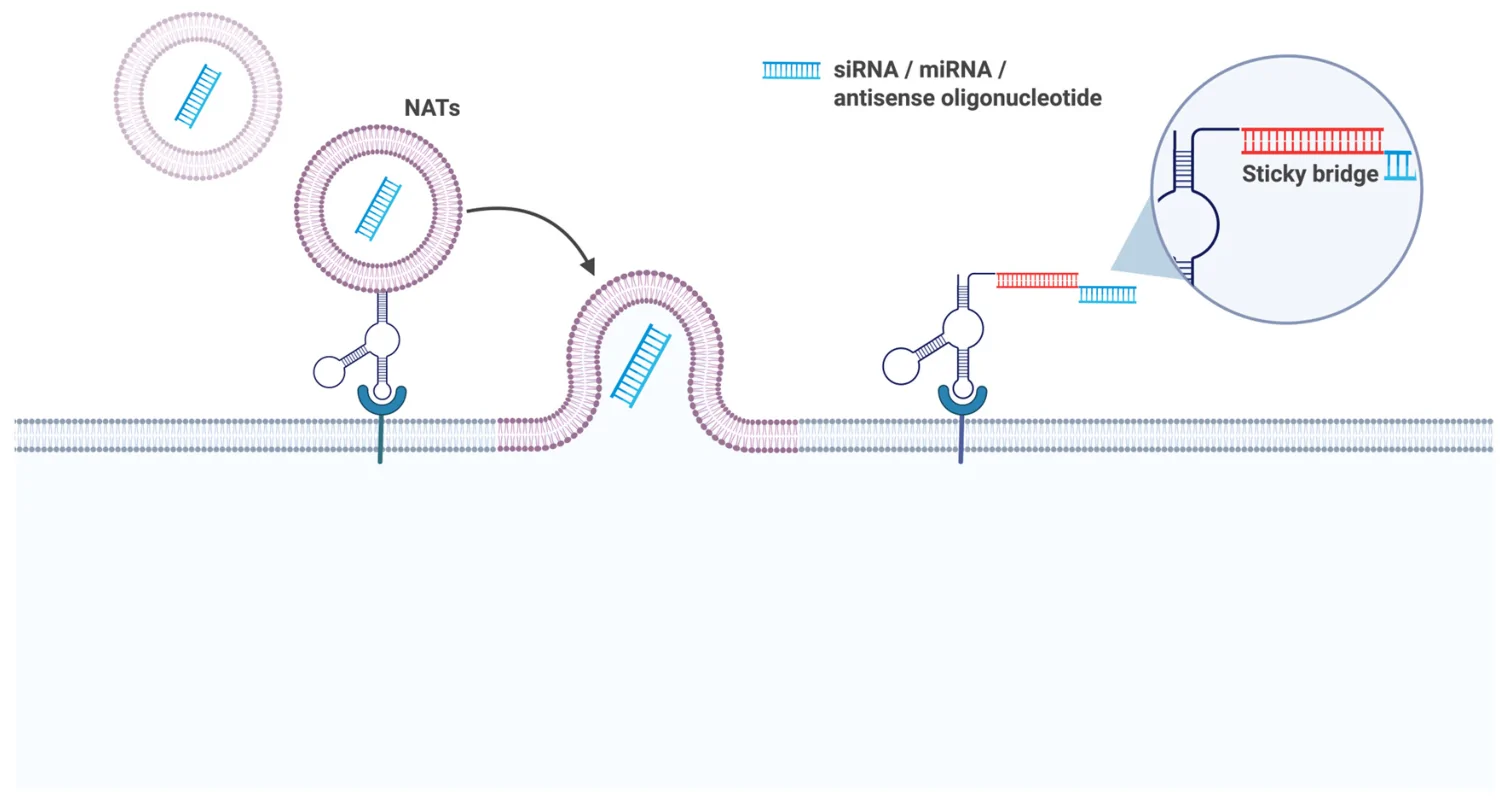
Illustration of two aptamer-based strategies for nucleic acid therapeutic (NAT) delivery in glioblastoma. (i) Covalent conjugation of an aptamer to a therapeutic nucleic acid (e.g., siRNA, miRNA, or antisense oligonucleotide), forming a linear chimera capable of selective cell targeting. (ii) Encapsulation of NATs within nanoparticles functionalized with surface-bound aptamers, generating Ap-NP) complexes. This configuration enhances BBB penetration and receptor-specific delivery. Directional arrows highlight the dual advantages of both systems: improved tumor selectivity and effective BBB crossing. Created in BioRender. Colasante, M. (2026) https://BioRender.com/t3rehd9 (accessed on 14 September 2026).

In GBM, aptamers offer a key advantage by facilitating travel through the BBB and enabling efficient intracellular delivery of therapeutic nucleic acids. Two main strategies dominate aptamer-mediated NAT delivery: direct covalent conjugation of aptamers to therapeutic nucleic acids, and incorporation of NATs into nanoparticles functionalized with aptamers to generate aptamer–nanoparticle (Ap–NP) complexes [111], (Figure 2).

Aptamer–siRNA conjugates (AsiCs) have attracted considerable attention as efficient vehicles for siRNA delivery. Their construction employs several conjugation strategies, including covalent linkage and annealing mediated by complementary sticky-bridge sequences. Following cellular internalization via endocytosis, the siRNA component becomes accessible to the RNA interference (RNAi) machinery, thereby enabling targeted gene silencing. Over the past decade, multiple aptamers have been exploited for siRNA delivery in glioblastoma models (Table 4).

STAT3-targeting siRNAs have been delivered using the PDGFRα-binding PDR3 aptamer and the PDGFRβ-specific Gint4.T aptamer, both effectively suppressing STAT3 activity in GBM cells [55,112]. Fei et al. developed a Gint4.T–HDGF siRNA AsiC that reduced GBM cell aggressiveness in vitro and significantly inhibited tumor growth and improved survival in vivo [113]. The BA aptamer, targeting U87-EGFRvIII cells, was similarly coupled to biotinylated c-Met siRNA via streptavidin, producing strong c-Met downregulation, increased apoptosis, and marked growth inhibition (*p* < 0.01) [44]. Other examples include an αvβ3-targeting aptamer linked to an siRNA against EEF2 and the A15 aptamer for CD133+ GBM stem cells conjugated to a Survivin siRNA [114,115]. siRNAs can also be delivered through cationic liposomes or composite and silica nanoparticles [113,114,116].

Survivin (BIRC5) is a key regulator of tumor cell survival and is highly expressed in GBM [117,118]. As an IAP family member, it inhibits apoptosis and promotes uncontrolled proliferation [108] and its overexpression correlates with poor prognosis and resistance to chemo- and radiotherapy [119]. Consequently, survivin inhibition, via siRNAs, small-molecule inhibitors, or targeted delivery platforms such as aptamers, is being explored to enhance treatment sensitivity [117,120,121,122]. A 2021 study used a DNA tetrahedron (TDN) to deliver Survivin siRNA into glioma cells; functionalization with the AS1411 aptamer (AsTDN-R) improved targeting, uptake, and gene silencing, leading to increased apoptosis and reduced survivin expression in U87 cells [123].

MiRNAs also play central roles in GBM biology [124,125] showing context-dependent dysregulation that affects angiogenesis, proliferation, and apoptosis. They can act as oncogenic or tumor-suppressive molecules, making them attractive NAT targets through anti-miRs, antisense oligonucleotides, or miRNA mimics. Frequently implicated miRNAs include miR-7, miR-34a, miR-128, miR-10b, miR-21, and miR-93 [126,127,128,129].

**Table 4:** Representative aptamer–NAT conjugates developed for glioblastoma therapy, detailing aptamer sequences, delivery strategies, and molecular mechanisms of action for siRNAs, anti-miRs, and survivin -targeting constructs.

Therapeutic Cargo	Aptamers	Delivery Strategy	Mechanism	References
siRNA	Gint4.T (anti-PDGFRβ) BA (anti c-Met) ανβ3 (anti EEF2) PDR3 (anti PDGFRα) A15 (anti CD33) AS1411 (anti nucleolin) SL1 (anti c-Met) TfR aptamer (anti TfR)	Direct aptamer–siRNA conjugation; nanoparticle system, modular nanostructures	Internalized siRNA is processed by RNA interference machinery, leading to selective degradation of target mRNA in vitro and in vivo models.	[33,34,43,54,60,113,114,115,116]
Survivin siRNA	A15 AS1411	Cationic liposomes, DNA tetrahedron nanostructures	Aptamer conjugates or TDN inhibit survivin expression, blocking glioma anti-apoptotic pathways and inducing tumor cell death as demonstrated in experimental nanostructure scaffolds.	[117,118,119,120,121,122,123]
anti-miR and miRNA mimics	GL21.T aptamer (GL21.T-10b-222) Gint4.T 40L A40s	Direct aptamer–miRNA and anti-miR conjugation	Internalized miRNAs regulate gene expression by binding target mRNAs, affecting tumor processes such as proliferation, invasion, and drug resistance. Anti-miRs sequester overexpressed oncogenic miRNAs.	[124,125,126,127,128,129]

Aptamer-mediated delivery of RNA therapeutics can rely on covalent linkers or non-covalent formats such as sticky-end annealing or streptavidin–biotin assembly. Aptamers can be attached to either strand of siRNAs, miRNAs, or anti-miRs. After endocytic uptake, miRNAs suppress target mRNAs, while anti-miRs neutralize oncogenic miRNAs.

Several proof-of-concept studies support this strategy. The GL21.T–anti-miR-222 chimera inhibited AXL signalling and reduced miR-222 levels, suppressing tumor growth in vivo [61].

GL21.T and Gint4.T have also been used to deliver miR-137 and anti-miR-10b to AXL+/PDGFRβ+ GSCs, inducing differentiation and impairing proliferation and spheroid formation [71].

Using cell-SELEX, Affinito et al. identified EphA2-binding aptamers (40L and A40s) selective for GSCs; A40s–miR-34c and A40s–anti-miR-10b chimeras modulated gene expression and reduced GSC viability [36].

A major advance is aptamer-directed suicide gene therapy. Di Leandro et al. (2021) engineered the APTSAP plasmid, placing the AS1411 aptamer upstream of the saporin gene to target nucleolin-overexpressing GBM cells. APTSAP induced strong cytotoxicity in U87 cells, while a catalytically inactive mutant (APTSAPKQ) was ineffective. Delivery via PEI poly transferrin receptor transfection and apoptosis, and microscopy suggested AS1411 adopts G-quadruplex structures that facilitate nucleolin-mediated uptake [130].

### 3.3. Aptamers Applications to Overcome Resistance to Standard Chemotherapy

Resistance to TMZ remains a major barrier to effective GBM therapy, prompting the development of innovative delivery systems to enhance chemosensitivity. Recent studies highlight the potential of aptamer-functionalized nanocarriers to overcome pharmacological and microenvironmental constraints that limit TMZ efficacy [57]. The following studies exemplify the application of specific aptamer-functionalized systems in overcoming these challenges:

- Tetrahedral framework DNA nanoparticles (TFDNs): Fu et al. (2019) engineered tetrahedral framework DNA nanoparticles (TFDNs) capable of encapsulating TMZ, thereby protecting the drug from rapid degradation and enabling controlled release. In GBM cell models, TMZ-loaded TFDNs improved intracellular delivery and induced higher rates of apoptosis compared with free TMZ. In vivo, the nanocarrier demonstrated efficient tumor localization, enhanced growth suppression, and reduced systemic toxicity, underscoring the promise of DNA nanostructure as delivery vehicles for TMZ with potential clinical translation [57].
- Glioma-targeting aptamer: Building on this concept, Zeng et al. (2023) developed an aptamer-guided nanoplatform integrating chemotherapy and photothermal therapy. This liposomal system, functionalized with a glioma-targeting aptamer, co-encapsulated TMZ and the photothermal agent IR780, enabling active tumor targeting across the BBB; the combined approach alleviated intratumoral hypoxia, increased TMZ sensitivity, and modulated the tumor microenvironment [131].
- Gint4.T–siHDGF chimera: Fei et al. (2024) demonstrated that mesoporous silica nanoparticles capped with the aptamer–siRNA chimera Gint4.T–siHDGF, co-delivering TMZ while silencing HDGF, achieved synergistic antitumor effects [113].
- Anti-CD133 aptamer: To target GSCs and reduce chemoresistant subpopulations, Smiley et al. employed anti-CD133 aptamer-decorated nanoparticles for co-delivery of TMZ and RG7388, an MDM2 inhibitor [132]. Dual drug loading into the hydrophobic core enables simultaneous delivery of agents with distinct mechanisms of action, enhancing therapeutic potency. Similarly, exosome-based platforms have been explored for combinatorial therapy: exosomes loaded with TMZ and O6-Benzylguanine (O6-BG) and decorated with anti-angiopep-2 peptide and anti-CD133 RNA aptamer (EXO-An2-Apt) improved survival by approximately 44% compared with free drug treatment in orthotopic GBM models. Histopathological analysis revealed minimal systemic toxicity in EXO-An2-Apt-treated mice, whereas free TMZ and O6-benzylguanine induced significant pulmonary and cardiac damage [133].
- A15 aptamer: In U87MG cells and GSCs, aptamer-functionalized exosomes achieved substantial intracellular accumulation, resulting in marked growth inhibition. Due to specific A15 aptamer binding, treated cells exhibited ~67.8-fold higher drug uptake and increased apoptosis compared with free TMZ and O6-BG [134].

DNA damage repair pathways are upregulated, and apoptosis is dysregulated by GSCs to develop TMZ resistance despite TMZ’s proven survival advantage. Because single-drug methods have failed to show any prognostic improvement during the past 15 years, new approaches, including multimodal combination therapies and methods of targeting delivery of drugs directly against cancer stem cells are urgently needed [135] in addition to CD133, GSCs express other biomarkers that may be targeted including Nestin, CD95, GFAP, and CD44 [136]. Recent research has demonstrated that the AC133 epitope on CD133 is a more specific marker for cancer stem cells (CSCs) [137]. During CSC differentiation, the AC133 epitope is sequestered and is thus only present in the undifferentiated form, and this is an important opportunity to selectively target undifferentiated CSCs. Given the difficulty in producing antibodies to the AC133 epitope, aptamers are being developed instead due to their capacity to conform to any three-dimensional shape and replicate the target ligand’s active binding site [115].

Previously described for its targeted delivery design, the TMSN@siHDGF-Gint4.T system also acts as a potent chemosensitizer. By silencing HDGF, it significantly enhances TMZ-induced cytotoxicity and apoptosis in GBM cells, amplifying therapeutic efficacy beyond its targeting function [113].

### 3.4. Aptamers and the Blood–Brain Barrier (BBB)

One of the principal challenges in GBM therapy is effective drug delivery across the BBB. Aptamers can exploit transcytosis-related receptors to facilitate BBB penetration, and recent efforts have focused on characterizing RNA aptamers capable of traversing the human BBB:

- hBS01–hBS06 (BBB-homing aptamers): Choi et al. employed an MPS-based SELEX platform that recapitulates the structural and functional complexity of the human BBB using iPSC-derived brain microvascular endothelial cells, astrocytes, and pericytes. This approach yielded a panel of BBB-homing aptamers (hBS01–hBS06), among which hBS01 demonstrated superior penetration and targeting capacity. Mechanistic studies revealed that hBS01 mediates protein cargo transport across the BBB via clathrin-dependent endocytosis, achieving enhanced uptake in endothelial and brain cells without compromising barrier integrity. In vivo validation confirmed efficient brain enrichment, positioning MPS-SELEX as a promising strategy for identifying clinically relevant aptamers for CNS therapeutics [68].
- GMT8 and Gint4.T (GTG system): Aptamer-functionalized nanostructures have also been leveraged to co-deliver chemotherapeutics and overcome BBB limitations. For instance, paclitaxel was incorporated into tetrahedral framework nucleic acids (tFNAs) conjugated with two aptamers, GMT8 and Gint4.T, to form Gint4.T–tFNA–GMT8 (GTG), which targets PDGFRβ and U87MG cells. In vitro, BBB models demonstrated efficient GTG uptake by U87MG and bEnd.3 cells, while paclitaxel-loaded GTG exhibited enhanced anti-glioma activity, inducing apoptosis and suppressing invasion, migration, and proliferation [61].
- HG1-9 (ApDC): Similarly, a transferrin receptor-targeted aptamer–drug conjugate (ApDC), HG1-9–MMAE, displayed potent cytotoxicity in U87MG-Luc2 cells and significant antitumor efficacy in orthotopic GBM models [58].
- AS1411 aptamer: A microemulsion platform incorporating the AS1411 aptamer and hyaluronic acid was developed to co-deliver shikonin and docetaxel (AS1411/SKN&DTX-M) for glioma treatment. This ~30 nm formulation demonstrates efficient BBB penetration and selective targeting of glioma cells overexpressing CD44 and nucleolin. Under acidic and hyaluronidase-rich conditions, the microemulsion rapidly released its payload, enhancing cytotoxicity, cellular uptake, and apoptosis in U87 cells, while suppressing CSC-like spheroid formation via CD133 downregulation. In vivo, AS1411/SKN&DTX-M accumulated in glioma-bearing brains and significantly inhibited tumor growth, yielding the longest survival among tested regimens [74]. In intracranial glioma models, angiopep-2 facilitated BBB penetration, enabling systemic administration. Similarly, PLGA-based nanoparticles conjugated with AS1411 (Ap–PTX–NP) demonstrated nucleolin-mediated uptake, prolonged circulation, and potent anti-glioma activity in C6 xenograft and intracranial models [138].

Additional strategies exploit aptamer-guided, stimuli-responsive delivery systems:

- ATP-binding DNA aptamer: An et al. designed a LAT1 substrate-based platform for doxorubicin (DOX) delivery using an ATP-binding DNA aptamer and a glutathione-responsive release mechanism [64]. The aptamer–cDNA scaffold intercalated DOX within GC-rich regions, enabling ATP-triggered drug release exclusively in tumor cells, while sparing normal tissues. In vitro studies confirmed tumor-selective DOX release confirmed the approach for precision chemotherapy. Paclitaxel (PTX), despite its established anticancer efficacy, suffers from poor solubility and systemic toxicity, prompting exploration of aptamer-functionalized nanoparticles for targeted delivery [59,139].
- A15 aptamer: Sun et al. developed a dual-modified cationic liposome incorporating angiopep-2, PTX, survivin siRNA, and the A15 aptamer targeting CD133+ GSCs [114]. This construct selectively induced apoptosis in GSCs, promoted differentiation into non-stem phenotypes, and reduced TMZ resistance in U251 cells. Given the heterogeneity of BBB integrity in gliomas, strategies combining tumor targeting with vascular modulation offer enhanced therapeutic precision. For example, AS1411-modified PTX nanoparticles exemplify this dual-targeting approach, reducing tumor burden and improving survival in preclinical models.
- U2 DNA aptamer: Peng et al. further advanced aptamer–nanoparticle systems by conjugating the U2 DNA aptamer to gold nanoparticles (AuNPs) via thiol chemistry. In U87-EGFRvIII cells, U2–AuNP complexes suppressed EGFRvIII phosphorylation within 24 h, inhibiting invasion and proliferation. In vivo, aptamer signals were detected in glioma-bearing brains 24 h post-administration, confirming BBB penetration and tumor-specific delivery [140].

### 3.5. Aptamers in Overcoming Resistance to Radiotherapy

GBM is a highly treatment resistant brain cancer, largely due to its poor response to radiotherapy [141], which drives recurrence and the dismal prognosis of affected patients. A major contributor to this resistance is the presence of GSCs, which efficiently repair DNA damage and survive radiation exposure. Tumor microenvironmental factors—particularly hypoxia and activation of PI3K/AKT and NF-κB pathways, further promoting apoptosis escape and sustained proliferation, complicating therapeutic control. Enhancing GBM radiotherapy therefore requires strategies that suppress these protective mechanisms, and the use of radiosensitizers represents a promising approach to amplify radiation induced cytotoxicity [142]. By increasing tumor cell death while limiting damage to surrounding tissues, radiosensitizers can significantly improve clinical outcomes [143]. Aptamer based conjugates with nanoparticles, targeted proteins, or other molecular agents offer an effective means to potentiate radiotherapy. Their coupling with small molecules, peptides, or immune modulating compounds enables multifunctional interventions that disrupt key pathways involved in tumor growth and radioresistance [143]. Among these, aptamer nanoparticle systems, particularly gold or silver based, are emerging as powerful radio sensitizing platforms. Li et al. developed PEGylated gold–silver core–shell nanoparticles (Ag@Au) functionalized with the GMT8 aptamer, exploiting the intrinsic radio sensitizing properties of both metals. GMT8 Ag@Au displayed enhanced glioma targeting and retention compared to unmodified systems, entering cells via clathrin mediated endocytosis and micropinocytosis. This delivery strategy yielded an SER of 1.62 and significantly prolonged survival in glioma bearing mice when combined with radiotherapy [144].

Similarly, Kan et al. engineered PEG Ag@AuNPs (PSGNPs) functionalized with TfRA4 and/or DNA1 aptamers. These TDSGNPs crossed the BBB, accumulated efficiently in tumors, increased apoptosis, and extended median survival in mouse models [145]. Zhao et al. proposed another radio sensitizing platform using silver nanoparticles functionalized with the nucleolin binding aptamer AS1411 and the P-gp inhibitor verapamil blocked P-gp mediated efflux, enhancing intracellular nanoparticle retention, while AS1411 enabled selective glioma uptake. A 19:1 mixture of AgNPs@BSA AS and AgNPs@BSA AS VRP optimized targeting and efflux inhibition. By promoting ROS generation and inhibiting thioredoxin reductase (TrxR), a key enzyme in radioresistance, this dual functional system markedly increased radiosensitivity in vitro and in vivo [146].

Among aptamers listed in Table 1, U2 exemplifies multimodal therapeutic potential. In vitro, U2 enhances radiosensitivity of U87 EGFRvIII cells by increasing apoptosis, reducing migration and invasion, and impairing post radiation DNA repair through decreased phosphorylation of major repair effectors, ultimately reducing proliferation and increasing cell death [32].

Targeted inhibition of key signaling cascades, including (1) PI3K/Akt, (2) MAPK, and (3) NF-κB, also improves susceptibility to radiation induced apoptosis [70]. Suppressing PI3K/AKT disrupts downstream regulators such as FOXO and mTOR, weakening pro survival signaling. Aptamers can similarly inhibit the MAPK pathway [147] interfering with cell cycle and stress response regulation, and downregulating NF-κB-dependent anti apoptotic proteins while enhancing pro apoptotic signaling [148].

Overall, radio sensitization strategies aim to increase ROS production and modulate the tumor immune microenvironment, and aptamers can be effectively employed alone or in combination with these approaches.

### 3.6. Aptamers and the Tumor Microenvironment

The microenvironment of GBMs is known to be hypoxic, acidic, and consists of many immunosuppressive cells that contribute to treatment resistance. Therefore, it is possible to design aptamers that will specifically target and neutralize molecules within the tumor microenvironment; the following research highlights the application of specific aptamers in targeting these microenvironmental factors:

- L-RNA aptamer NOX-A12: As previously described, this aptamer targets CXCL12, a chemokine central to tumor growth and immune evasion [148] and has shown preclinical potential by disrupting tumor vasculature and enhancing responses to combined therapies [51].
- Anti-VEGF Aptamer (Pegaptanib): Targeting vascular endothelial growth factor (VEGF), it plays a key role in trying to influence the GBM microenvironment, which is known for abnormal blood vessel formation often triggered by low oxygen levels. The RNA aptamer Pegaptanib binds selectively to the VEGF165 isoform, preventing it from interacting with VEGF receptors found on endothelial cells. By neutralizing VEGF in the tumor’s surroundings, this aptamer-based method seeks to make the blood vessels more normal, lower the pressure of fluid between tissues, and, hopefully, improve how well chemotherapy drugs can reach the tumor [53,54].
- GB-10 aptamer: Tenascin C is an extracellular matrix protein that is markedly overexpressed in the glioblastoma microenvironment, where it supports tumor cell adhesion, migration, and angiogenesis. The DNA aptamer GB-10, identified through SELEX, binds Tenascin C with high affinity in GBM tissues. Several studies indicate that GB-10 can be used to deliver radionuclides or nanoparticles directly to the tumor stroma, minimizing exposure of healthy brain tissue and taking advantage of the protein’s abundance within the GBM altered matrix [47].
- TTA1: The RNA aptamer TTA1 binds the fibrinogen like domain of Tenascin C with high affinity. In preclinical GBM models, TTA1 shows rapid accumulation within the tumor and clears efficiently from the bloodstream, features that make it well suited for molecular imaging and for delivering therapeutic radioisotopes directly to the tumor stroma. By targeting this highly overexpressed component of the GBM microenvironment, TTA1 based platforms can concentrate therapeutic payloads within the tumor mass while limiting exposure to the surrounding healthy brain tissue [48].

### 3.7. Aptamers as Adjuvants in TTFields Based Therapy

TTFields (Tumor Treating Fields) use low-intensity electric fields (1–3 V/cm) at intermediate frequencies (100–300 kHz) to disrupt microtubule polymerization and spindle assembly, thereby preventing mitosis. In newly diagnosed GBM patients, the FDA approved TTFields in 2015 based on clinical data demonstrating superior 5-year survival in patients receiving both TTFields and temozolomide versus chemotherapy alone [149]. Despite their efficacy, there is still scientific uncertainty surrounding the mechanism of how TTFields work as an anticancer treatment, and a notable percentage of patients will develop resistance to TTFields after treatment. Researchers are now exploring whether combining TTFields with other therapies could improve clinical outcomes for patients. Researchers are exploring the development of aptamers that target GBM-specific biomarkers to deliver drugs and enhance the cytotoxicity of TTFields. For example, aptamers that bind EGFRvIII or nucleolin could deliver agents that alter the properties of cell membranes or induce apoptotic pathways to increase the amount of cellular damage caused by exposure to TTFields. Multi-serotype aptamer constructs could present multiple sites of attachment thereby increasing the likelihood that TTFields sensitizing agents would have access to their intended intracellular targets [150].

A further strategy utilizes aptamers to deliver immune modifying molecules (e.g., checkpoint blockade agents or cytokine-encoding sequences): it has been shown that TTFields may activate the immune response, indicating that aptamer enhanced immune function may act in concert with the effects produced by the TTFields. There is strong evidence to suggest that the combination of the ability of TTFields to alter the organization of cellular structure and the membrane with the ability of aptamers to specifically deliver a payload may lead to a synergistic therapeutic effect [151].

As of now, hardly any research has been done to investigate the effectiveness of combining aptamers with TTFields in a clinical trial setting; thus, significant gaps remain our current knowledge about this combination therapy. Nonetheless, there is an extensive body of evidence to support biological plausibility for these two treatment modalities working synergistically together; in particular, TTFields alter the cellular microenvironment to allow for enhanced uptake and transport of the intended therapeutic agent, while also creating a microenvironment in which aptamer-bound tumor cells are more susceptible to the effects of the electric field produced by TTFields. The results of future preclinical studies should provide more definitive evidence of the interactions between TTFields and aptamers in preclinical animal models of GBM, including investigations of the optimal timing and dosing regimen for using aptamers in conjunction with TTFields and determining which particular subtypes of GBM are most likely to respond favorably to this combination of therapies [148].

**Figure 3:**
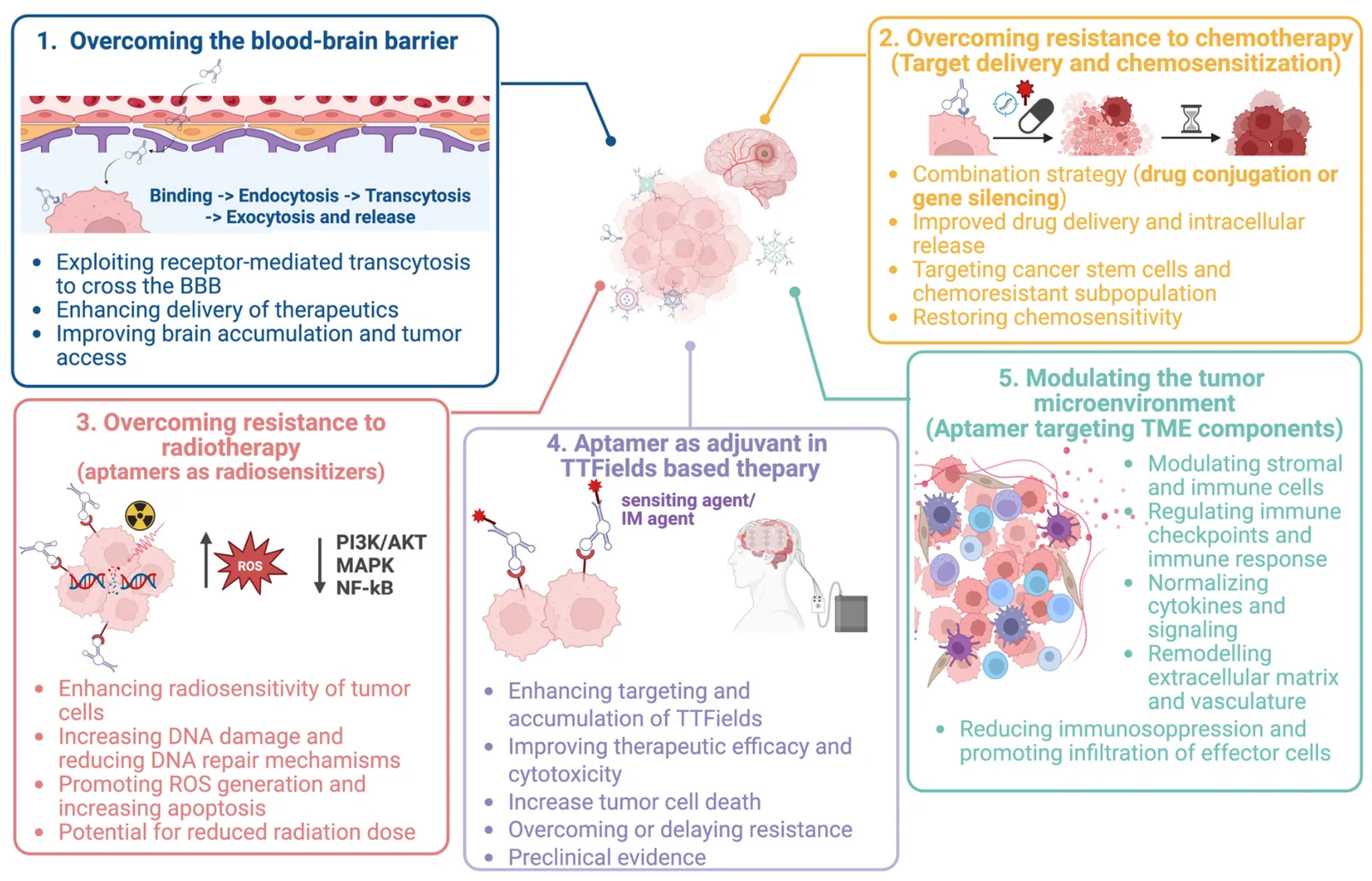
Aptamer application in GBM therapy. Aptamers can be exploited to facilitate BBB penetration, enhance chemosensitivity and radiosensitivity, modulate the tumor microenvironment and support the therapeutic effect of TTFields. Their integration with targeted delivery systems and combination therapies provides a versatile approach to improving treatment efficacy and tumor selectivity. Created in BioRender. Colasante, M. (2026) https://BioRender.com/t3rehd9 (accessed on 14 September 2026).

The main therapeutic applications of aptamers in GBM are summarized in Figure 3.

### 3.8. Criticisms and Perspectives

Although multivalent aptamer platforms and aptamer-based conjugates offer an attractive strategy to integrate tumor targeting with chemotherapy, radiotherapy, nucleic acid therapeutics, and BBB transport, increasing molecular complexity also introduces important translational challenges as to be confirmed in real clinical environments to fully match the spatial heterogeneity, immune environment, vascular architecture, and treatment resistance in human GBM. Furthermore, the therapeutic contribution of the aptamer itself is not always clearly distinguishable from that of the nanoparticle, therapeutic cargo, or additional targeting ligand, making it difficult to determine which component is primarily responsible for the observed benefit. Multicomponent systems may furthermore also raise concerns regarding reproducible manufacturing, batch-to-batch variability, pharmacokinetics, long-term toxicity, and regulatory approval. This issue is particularly relevant for combinations with TTFields, for which the biological rationale is intriguing, but direct experimental evidence for aptamer-mediated synergy remains scarce. Consequently, future investigations should mainly focus on systematic head-to-head comparisons, appropriate mechanistic controls, orthotopic and patient-derived models, and quantitative assessment of whether the added complexity of multivalent systems produces a clinically meaningful advantage over simpler therapeutic platforms.

## 4. Bispecific Aptamer–Targeting Entities (BATEs): The Latest Evolution in Aptamer Engineering

Recent advances in bivalent aptamer constructs (BATEs), such as CL4–Gint4 or AS1411/GS24, have demonstrated the feasibility of simultaneously engaging distinct receptors (e.g., EGFRvIII and PDGFR), a strategy designed to address intra-tumoral heterogeneity [57,103]. Multivalency can enhance functional avidity, promote receptor cross-linking, and in specific receptor-targeting designs has also been proposed to enhance receptor-mediated transcytosis across the BBB, thereby improving distribution to tumor parenchyma while maintaining specificity [23,92,148,152].

BATEs (Bispecific Aptamer–targeting Entities) have emerged as a promising strategy with significant potential for treating complex, heterogeneous tumors such as GBM. Unlike monovalent aptamers, which typically engage a single epitope, BATEs are engineered to simultaneously bind multiple molecular targets, thereby enhancing avidity, specificity, and functional versatility [153]. This multi-target capability can improve discrimination between tumor and normal cells and may promote receptor clustering, with the potential to facilitate synergistic modulation of downstream signaling pathways. The resulting cooperative binding effect produces an avidity exceeding that predicted from the individual monovalent interactions, prolonging receptor engagement and increasing therapeutic precision toward tumor phenotypes expressing multiple markers [154,155].

Furthermore, receptor cross-linking mediated by BATEs can augment cellular uptake and retention, which in some systems has been associated with improved intracellular delivery and therapeutic activity against complex biological targets. The design of multivalent aptamers requires precise spatial organization of monovalent subunits to optimize avidity and functional performance; this architecture depends on the nature of interactions between aptamer/target and the therapeutic application [156]. While direct covalent linkage of aptamer units is conceptually simple, it is rarely employed due to structural constraints. Instead, flexible linkers, either nucleotidic (e.g., poly-T or poly-A sequences) or non-nucleotidic (e.g., polyethylene glycol PEG), are commonly used to prevent unintended secondary structures and minimize steric hindrance between adjacent aptamer domains. Linker length and flexibility critically influence receptor clustering and functional avidity, underscoring their importance in multivalent aptamer design [156].

Dual-targeting strategies exemplify the therapeutic potential of multi-specific aptamers; a frequent method combines one aptamer for tumor-specific receptor recognition with another that facilitates internalization via receptor-mediated endocytosis, thereby improving intracellular delivery and therapeutic efficacy [157,158].

This concept extends to BBB-targeted designs, which address a major obstacle in GBM therapy. Bifunctional aptamers have been designed to bind a tumor-specific surface marker and a BBB transport receptor, enabling transcytosis of therapeutic payloads into the CNS [62,147]. For example, a bifunctional construct linking the EpCAM-targeting SYL3C aptamer (truncated to 17 nucleotides) with a 14-nt transferrin receptor (TfR) aptamer was shown to cross the BBB in a mouse model through TfR-mediated transcytosis while exerting anti-metastatic effects through EpCAM targeting [63]. Beyond direct tumor targeting, aptamers can be designed to modulate host immune responses, offering an alternative to conventional immunotherapies such as monoclonal antibodies or vaccines. Aptamer-based immune modulators provide high target specificity, minimal off-target effects, and structural adaptability, making them attractive candidates for further preclinical and clinical investigations.

**Figure 4:**
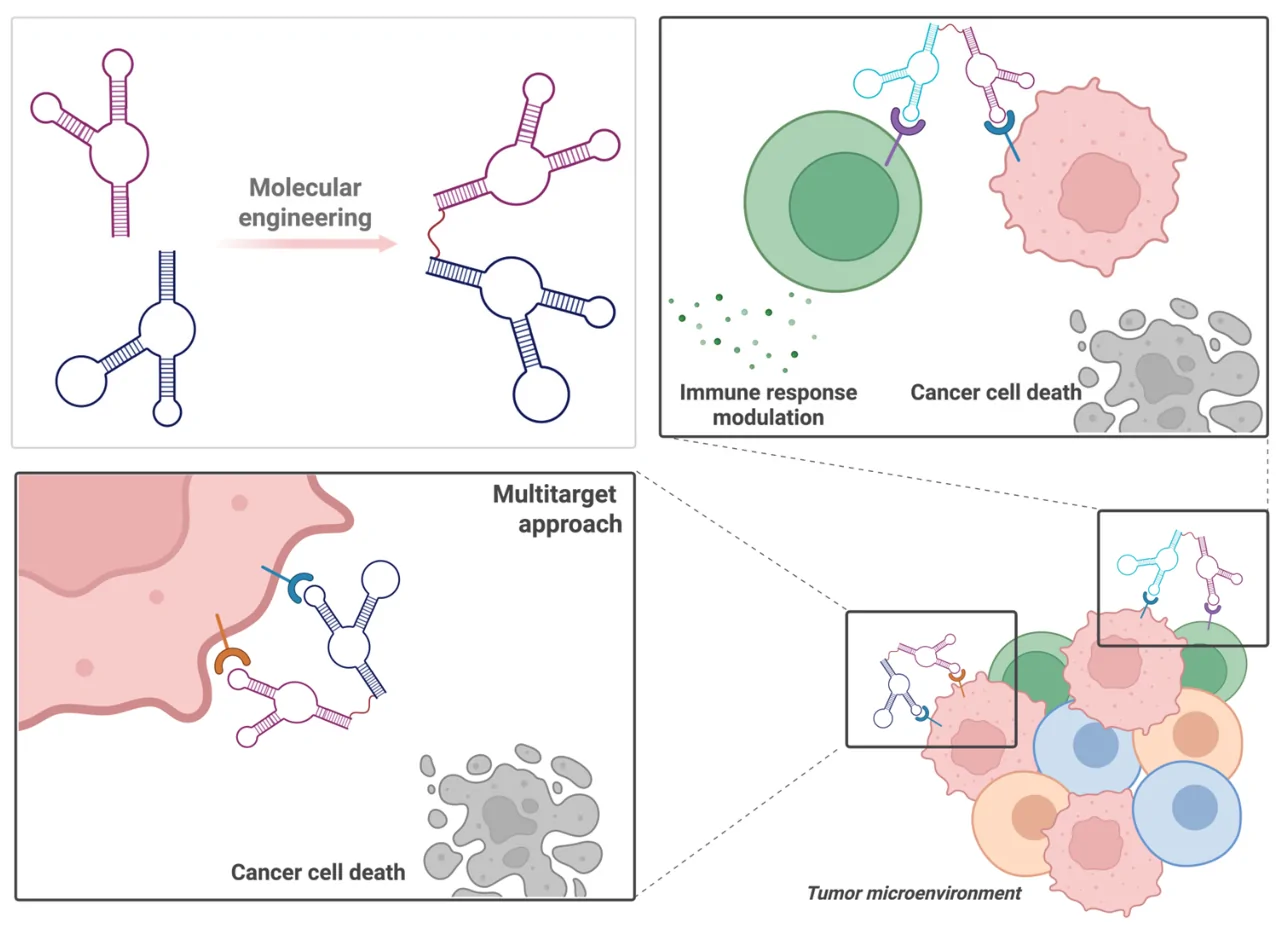
Schematic representation of a bispecific aptamer designed to enhance therapeutic precision in cancer immunotherapy. The construct combines an immune-cell targeting aptamer (red), specific for receptors such as CD3 or CD8, with a tumor-targeting aptamer (blue), directed against surface markers like nucleolin or EpCAM. Hybridization between the single-stranded regions of the two aptamers forms a duplex bridge, resulting in a functional bispecific aptamer. Created in BioRender. Colasante, M. (2026) https://BioRender.com/t3rehd9 (accessed on 14 September 2026).

To limit systemic toxicity, immune-modulatory aptamers are often conjugated to tumor-specific aptamers, thereby restricting their activity to malignant cells and enhancing safety profiles [62] (Figure 4).

Recent advances have introduced aptamer-based strategies targeting immune checkpoints as promising approaches for glioblastoma (GBM) therapy [159]. Among these, the co-stimulatory receptor 4-1BB (CD137) has emerged as a compelling target due to its role in enhancing antitumor immunity by promoting CD8^+^ T-cell survival, expansion, and differentiation into memory cells [160]. Systemic administration of anti-4-1BB antibodies has demonstrated potent antitumor effects in murine models, particularly when combined with vaccines or other immunotherapies [160,161,162]. However, antibody-based approaches are frequently associated with immune-related adverse effects, including polyclonal CD8^+^ T-cell activation and excessive production of pro-inflammatory cytokines such as IFNγ and TNF [163,164,165]. To mitigate these limitations, Schrand et al. developed a bispecific aptamer construct comprising a VEGF-targeting aptamer, leveraging VEGF abundance in the GBM microenvironment for localized delivery, and a 4-1BB aptamer to co-stimulate T cells. In high-grade glioma models characterized by PDGFRβ and STAT3 overexpression, VEGF–4-1BB aptamers significantly prolonged survival compared with controls in murine high-grade glioma models, demonstrating preclinical therapeutic potential and the ability to cross the blood–brain barrier in these experimental settings [166,167].

A similar bispecific approach was employed by Jun Wei and colleagues to target tumor-associated macrophages. Their construct combined the OPN-R3 aptamer, which binds osteopontin (OPN), a glycoprotein overexpressed in GBM-infiltrating immune cells and linked to poor prognosis, with a 4-1BB aptamer to enhance CD8^+^ T-cell activation. This dual-target system inhibited migration of M0 and M2 macrophages while stimulating cytotoxic T-cell activity in preclinical studies, ultimately extending survival in GL261 intracerebral glioma models [168]. Additional bifunctional aptamer designs address the challenge of BBB penetration. One example is the TEPP construct, which integrates the EpCAM-targeting SYL3C aptamer (truncated to 17 nucleotides) with a 14-nt TfR aptamer. TEPP enables transcytosis across the BBB via TfR-mediated transport while selectively binding EpCAM-expressing tumor cells, thereby combining CNS delivery with anti-metastatic activity [169].

Beyond therapeutic applications, aptamer-based platforms have been adapted for diagnostic purposes. Wang et al. (2023) developed a multivalent aptamer nano-scaffold-based electrochemical cytosensor (MAS-cytosensor) for detecting circulating tumor cells (CTCs) undergoing epithelial–mesenchymal transition (EMT) in glioma. This system employs aptamers targeting EpCAM and cell-surface vimentin (CSV) to capture CTCs across EMT states, while the rigid DNA nano-scaffold optimizes probe density and orientation. The MAS-cytosensor demonstrated high sensitivity, detecting EMT-CTCs at concentrations as low as six cells per mL, and enabled viable recovery of captured cells for downstream culture, offering a valuable tool for monitoring metastatic progression and therapeutic response [170].

Hybrid and multivalent aptamer systems further expand the therapeutic landscape. Monaco et al. designed BODIPY@PNPs–Gint4.T, a PLGA-b-PEG nano-vector functionalized with the PDGFRβ-targeting aptamer Gint4.T, which achieved efficient delivery in intracranial GBM models, leveraging the heterogeneous permeability of the blood-tumor barrier (BTB)**;** however, the reliance on the enhanced permeability and retention (EPR) effect in GBM remains a subject of debate due to the high variability in vascular disruption [171].

Similarly, Saw et al. engineered aptamer-like peptide ligands targeting the extra-domain B (EDB) of fibronectin and conjugated them to PEGylated liposomes for RNA interference delivery, resulting in significant tumor suppression [172].

Hybrid constructs such as TGN–AS1411, which combines the BBB-penetrating TGN peptide with the nucleolin-binding aptamer AS1411, exemplify multifunctional designs that integrate transport and tumor targeting without increasing toxicity to normal brain tissue [92]. Collectively, these innovations underscore the versatility of aptamer-based architectures in overcoming biological barriers and achieving precision delivery in GBM therapy.

### Criticisms and Limiting Factors

BATEs represent a compelling approach to GBM heterogeneity because simultaneous recognition of multiple targets may increase avidity, cellular internalization, and tumor selectivity. However, whether bispecific or multivalent binding translates into superior therapeutic efficacy remains unclear. Receptor expression in GBM is highly heterogeneous and dynamic, so optimal target combinations may vary across patients, tumor regions, and disease stages. Moreover, receptor cross-linking may produce context-dependent effects on signaling pathways. The incorporation of multiple aptamer domains and linkers also introduces structural variables, including linker length, orientation, steric interference, and folding stability, which can affect in vivo performance. Importantly, most BATE studies remain preclinical, and BBB penetration in experimental models does not necessarily predict effective distribution within human infiltrative GBM. Therefore, future development should focus on pharmacodynamic validation, optimization of molecular architecture, rational target selection, and direct comparisons with monovalent or combinatorial approaches to determine whether bispecific constructs provide a meaningful therapeutic advantage.

## 5. Future Perspectives in Aptamer Design: Computational Acceleration of Discovery

**Figure 5:**
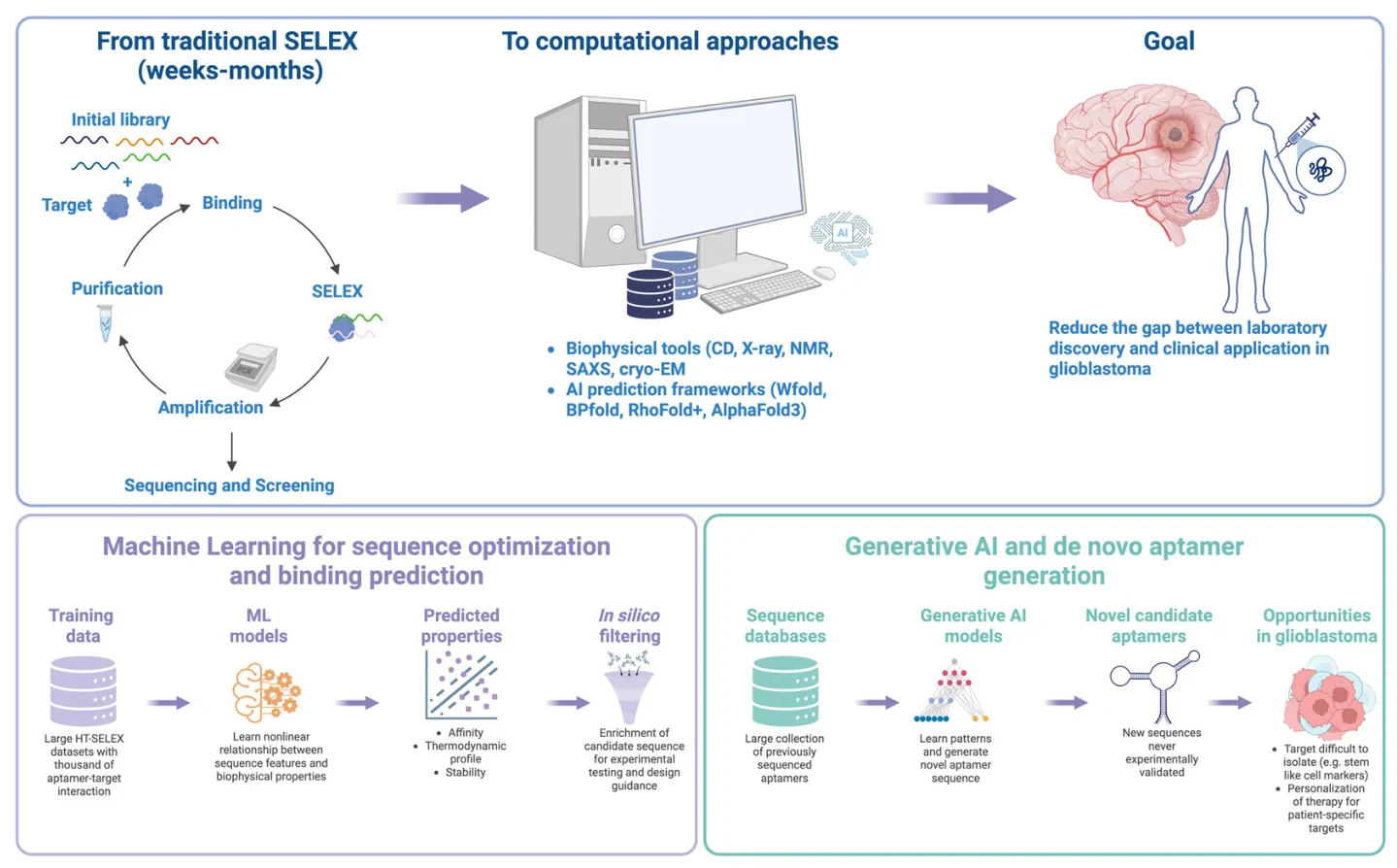
Schematic overview of the transition from conventional SELEX to computational and AI-driven approaches for aptamer discovery, optimization and de novo generation, with applications in Glioblastoma therapy. Created in BioRender. Colasante, M. (2026) https://BioRender.com/t3rehd9 (accessed on 14 September 2026).

Aptamer discovery has traditionally relied on SELEX (Systematic Evolution of Ligands by EXponential Enrichment), which is a highly time consuming process that typically requires multiple rounds of selection over several weeks or even months. The application of computational methods and machine learning algorithms in the field of aptamer screening is currently changing the whole engineering process, enabling rational design approaches for the development of aptamers for a wide range of biomedical applications, spanning neuro-oncology (e.g., glioblastoma), systemic cancers, infectious diseases, and neurodegenerative disorders. According to a recent review on this subject, made by Zhao et al., the affinity, specificity, and biostability of aptamers is clearly dependent on their three-dimensional structures [173]. Biophysical characterization tools, including circular dichroism (CD), X-ray crystallography, X-ray crystallography, Nuclear Magnetic Resonance (NMR), Small-Angle X-ray Scattering (SAXS), and cryogenic electron microscopy (cryo-EM), coupled with emerging AI-driven prediction frameworks, such as Wfold, BPfold, RhoFold+, and AlphaFold 3, have significantly expanded the understanding of aptamer target binding interfaces [174,175,176,177]. Incorporating structural motifs that perform a specific biological function in an experimental screening library (such as G-quadruplexes, circular molecules or three-way junctions) or utilizing a computer algorithms such as SMART-Aptamer, RaptGen and RhoDesign is a method to control high stochasticity and increase selection efficacy [178,179,180]. Post-SELEX optimization strategies, including stem-loop engineering, rational truncation, terminal stabilization (triple helices, chemical cross-linking), circularization, and rigid scaffold construction, further restrict conformational flexibility and enhance nuclease resistance. The convergence of generative AI models, structural databases, and rational design frameworks is expected to narrow the gap between laboratory-based aptamer discovery and clinical translation [173]. The overall transition from conventional SELEX to computational and AI-driven approaches for aptamer discovery and optimization is summarized in Figure 5.

### 5.1. Machine Learning for Sequence Optimization and Binding Prediction

The application of machine learning is proving to be a powerful approach for understanding how small changes in aptamer sequences affect binding affinity, stability, and interaction properties. Using large datasets generated through HT-SELEX (High Throughput SELEX), machine learning algorithms are trained based on the interaction between actual aptamers and their targets. Extensive training of these models allows them to identify nonlinear relationships between sequence features and biophysical properties such as the equilibrium dissociation constant (KD) and thermodynamic profile (∆G) [181,182]. For the design of specific aptamers for GBM treatment, predictive analysis can serve as an important computational filter to guide rational modifications in the design process.

Single-stranded RNA or DNA oligos are extremely sensitive to rapid nuclease digestion in serum, making chemical modifications necessary to improve their drug-like properties. Machine learning approaches can be used to predict the best positions for the incorporation of modifications, such as 2′-F pyrimidines or LNAs, into the aptamer backbone in order not to hinder the hydrogen bonding and van der Waals interactions which are the basis of the molecular recognition process [183].

Moreover, whereas traditional sequence based models cannot directly account for macroscopic pharmacokinetic processes, advanced multi-objective machine learning classifiers can simultaneously evaluate candidate sequences against structural features known to enhance receptor mediated transcytosis, enabling a more comprehensive assessment of in vivo performance.

This approach could facilitate the selection of sequences designed to interact with the BBB transcytosis pathways (transferrin or LRP1 receptor) or sequences that can undergo PEGylation, which are both important steps for GBM therapy [104,184]. Even though these computational predictions are purely probabilistic in nature and thus do not help to avoid the necessity of laboratory testing, they can serve as an extremely valuable mathematical enrichment procedure to reduce the overwhelming set of sequences to high probability candidates.

### 5.2. Generative AI and De Novo Aptamer Generation

In addition to improving the use of previously established sequences, generative AI has the potential to suggest entirely novel aptamer sequences predicted to bind specific targets as well. These types of models utilize existing information from large databases of previously sequenced aptamers and create new sequences that have never been experimentally validated before. For GBM, this means there are several new opportunities available for discovering novel aptamers utilizing targets that are challenging to isolate via traditional SELEX methods including certain specific antigens on glioblastoma stem cells and some immunosuppressive ligands (such as PD-L1 and LAG3) and certain mutations associated with tumors. Earlier generative machine learning algorithms, including conventional neural networks and early GANs, had clear shortcomings in aptamer generation. Severe restrictions imposed by available datasets made it difficult to generate completely new sequences and explore unique sequence classes. An improvement over these earlier approaches are represented by RaptGen, a variational autoencoder-based generative model that explores sequence space in silico by decoding latent secondary structure motifs to optimize aptamer folding geometries and target selectivity [179]. However, its main translational limitation lies in its dependence on static 2D structural representation, which often fails to predict 3D conformational shifts, serum stability, and off-target binding in vivo environments.

The current state of the art in the field is represented by newer generative algorithms, such as AptaDiff [185]. By leveraging information on key binding motifs, AptaDiff designs new sequences optimized for target interaction, with the potential to achieve both high binding affinity and structural stability [185]. The quantitative performance metrics of this model, its benchmark comparisons with the other outperformed aptamer models and the status of their translation are listed in the Supplementary Table S1.

### 5.3. In Silico SELEX and Computational Selection

In silico SELEX and computational aptamer engineering have been widely applied across medical biotechnology to accelerate the discovery of targeted therapeutic and diagnostic tools. By passing the physical screening of massive oligonucleotide libraries, in silico selection accelerates aptamer discovery, dramatically cutting time, costs, and the number of experimental rounds while streamlining the identification of high-affinity candidates. At the same time, the silico approach employs sequence analysis, structural prediction, molecular docking, molecular dynamics simulation, and machine learning algorithms, to filter out promising leads prior to their testing in the laboratory settings. The design workflow begins by predicting how the aptamer folds into its functional secondary and tertiary conformations using software such as Mfold, ViennaRNA, and RNAComposer. Subsequently, molecular docking algorithms like AutoDock Vina (version 1.2.0) and HADDOCK simulate the physical assembly of the aptamer–target complex, providing quantitative estimates of binding affinity and mapping the molecular interactions at the binding interface [186].

Computational approaches have played a critical role in GBM research by enabling the targeted design and structural optimization of aptamers against key oncogenic receptors, such as the mutant EGFRvIII and wild-type EGFR. Furthermore, molecular modeling and docking simulations have facilitated the rational development of aptamers targeting the TfR1 to mediate BBB transcytosis for effective drug delivery into central nervous system lesions [187,188].

However, recent advancements in artificial intelligence have further streamlined, the process using learning tools like AptaNet, which can accurately determine the interaction between aptamers and their targets simply based on sequence data [189]. A significant advance in this field is represented by RaptScore, a large language model (LLM)-based framework introduced in 2026 for aptamer evaluation [190]. The RaptScore algorithm encodes nucleic acid sequences into predict target binding affinity without being constrained by the traditional SELEX approach, which solely depends on sequence enrichment. This makes it possible to analyze new molecules and define their final length to identify the minimal binding site [190].

### 5.4. AI-Driven Prediction Frameworks for Aptamer Design

To overcome the experimental limitations of conventional SELEX, predictive AI frameworks are becoming increasingly important. Recent advances in deep learning enable the fully in silico design of aptamers through accurate prediction of molecular recognition and target-binding affinity, addressing key limitations of classical approaches, including high costs, lengthy experimental workflows, and incomplete exploration of sequence diversity.

Aptamer design computational methods are developing quickly, progressing from secondary structure prediction to complete 3D modeling [173,175,181]. RNA language models can predict three-dimensional conformations directly from primary sequences, while thermodynamics-based algorithms precisely map folding stabilities and intricate patterns like pseudoknots. Moreover, whole aptamer–target complexes can now be directly modeled using sophisticated structural frameworks such as AlphaFold 3, which capture key atomic interactions at the binding interface [177,191,192]. These technologies speed up candidate selection by estimating parameters such as binding affinity (KD) and free energy (∆G). However, computational predictions still require experimental validation due to the intrinsic dynamic complexity of biological systems [193]. The high false-positive rate during experimental validation is a significant translational bottleneck in silico aptamer discovery. This is caused by three main factors: (1) Structural Over-simplification, whereby static models and tools like AlphaFold3 fail to capture the conformational flexibility of aptamer loops or correlate ipTM scores with true binding energy; (2) False Affinity Predictions, whereby favorable stereochemical fits computationally identified do not necessarily translate into functional activity; and (3) Biological Complexity, as models are unable to account for macromolecular crowding, conformational shifts, and nuclease degradation in living systems. This translational challenge is highly relevant to GBM research, where a significant portion of computationally derived aptamer candidates has not yet undergone comprehensive experimental validation in patient-derived models [194]. Conventional in silico target screening approach typically considers molecules target as a static biomolecule, which leads to a major drawback of the omission of important dynamism associated with the pathogenesis of GBM, which includes intratumoral heterogeneity, plasticity of GSCs, subclonal evolution, and pharmacokinetic restrictions of BBB [194].

To address these limitations, systems-biology and network-theoretic approaches have increasingly emphasized that glioblastoma should be interpreted as a complex adaptive system characterized by dynamic cell-state transitions, phenotypic plasticity, and attractor-like regulatory landscapes. Information-theoretic and network reconstruction frameworks have been proposed to identify key regulators governing glioblastoma evolution and differentiation trajectories, providing complementary tools to conventional molecular target identification. Such models may contribute to future computational pipelines aimed at prioritizing therapeutic targets and designing adaptive precision-medicine strategies in highly aggressive and heterogeneous GBM ecosystems. A comprehensive review on this issue is reported in [195,196].

Recent approaches merge systems biology and lead-conditioned generation to reduce the gap between computational predictions and the effective results observed in vivo. Non-linear network models, including percolation theory and attractor states, have been employed to improve the analysis of adaptative cancer signaling. In addition, lead-conditioned optimization allows restricting the parameters of the search to those regions where binders are located, thus reducing RMSD while preserving the important interactions in the vicinity of those entities such as hydrogen bonds and salt bridges. A comprehensive comparison mapping the performance characteristics, typical limitations and validation statuses of these emerging algorithmic families, alongside cancer complexity methods, is summarized in Supplementary Table S1.

In a recent paper, Qian et al. (2026) described POTFlow, the first lead peptide-conditioned flow-matching framework to be successfully validated in a GBM setting through both in vitro and in vivo experiments [197].

Although originally developed for therapeutic peptide design against ATP5A, a metabolically relevant GBM target characterized by aberrant surface exposure [198], its underlying mathematical architecture may be readily extended to address key challenges in aptamer discovery, particularly the generation of structurally and biologically plausible candidates while efficiently exploring the vast sequence and conformational landscape.

Instead of utilizing an unconstrained, unconditional search space, this framework implements a Coupled Conditional Flow Matching (C2FM) protocol. By extracting class-specific centroids from the secondary structure of a known lead sequence (mapping structural motifs such as helices, sheets, and loops via DSSP) and executing an intra-sample multimodal Optimal Transport (OT) policy, the model establishes shorter and more direct flow paths across distinct geometric spaces [197].

These localized constraints improve structural and biological plausibility during sample generation and enable the algorithm to outperform conventional unconditioned models, achieving a lower RMSD (1.66 Å) and a localized BSR of 87.01% [197].

Importantly, recent trends in neuro-oncology underscore that such computational optimizations must be treated merely as preliminary steps, requiring direct correlation with functional activity in preclinical experimental models [193].

Qian et al. addressed the limitations of in silico design through rapid validation in patient-derived models. Functional screening identified an optimized candidate (Candidate 4) that formed a high stable non-covalent complex with the target pocket through three hydrogen bonds and two hydrophobic interactions. Further in vitro experiments showed that the candidate demonstrated a favorable selectivity profile decreasing intracellular levels of ATP and viability in several patient-derived GBM lines (39 347, 99 567, and U87) and was less toxic to non-cancerous cells (<10%). In addition, therapeutic efficacy was assessed using an orthotopic patient-derived xenograft (PDX) mouse model to compensate for the impact of microenvironmental conditions. The administration of the drug (20 mg/kg) via subcutaneous osmotic pumps in an intracranial delivery model, led to a remarkable reduction in the growth of bioluminescent tumors throughout an 11-week observation period and significantly improved the survival of mice (*p*-value = 0.02). While future iterations in aptamer therapeutics must further integrate multi-objective predictors for blood–brain barrier permeation [182] and dynamic network feedback loops [183] the strategic integration of lead-conditioned generative flow matching with preclinical evaluation pipelines presents a cautious, step-by-step pipeline, emphasizing that in silico predictions must always remain subordinate to experimental validation [197].

## 6. Conclusions and Critical Perspectives on the Translation of Aptamer Therapies for Glioblastoma

While there has been considerable advancement in aptamer engineering, the translation gap in the field continues to be an ongoing problem, whereby although there have been very encouraging results from pre-clinical trials in the development of therapies against GBM, none of these have succeeded in yielding any clinical therapy for this type of malignancy. There exist various structural limitations that contribute to this problem that need to be addressed more fully. The first of these is the difficulty in delivering the therapy efficiently, effectively and reproducibly across the human BBB. Though the use of nanoparticles and receptor-mediated transcytosis, among other delivery methods, show great promise both in vitro and in animal models, they have all proved inadequate for human central nervous system delivery and clinical use. The extensive time and space variability inherent in GBM makes this an even more challenging process. An aptamer directed against only one specific target is not likely to be effective in tumor tissues where the receptor dynamics vary according to changing expression profiles, clonal variations and environmental plasticity. Multivalent and bispecific designs may offer solutions to some of the issues involved, but the greater complexity means that issues with pharmacokinetics, elevated immunogenicity (which remains scaffold- and modification-dependent) and additional regulatory hurdles may arise. This, in turn, makes the clinical development process difficult and raises questions regarding its scalability and economic viability relative to other options like antibodies, CAR-Ts and small molecules.

Limitations of pharmacokinetics are equally significant. Rapid excretion through kidneys and nucleic acid degradation due to nucleases still limiting the time during which therapeutic action occurs, despite the modification of backbone, PEGylation, or formation of the closed loop. Additionally, the suppressive environment of the tumor, including its hypoxic state, myeloid-derived suppressor cells, and malfunctioning cytokine signaling mechanisms, reduces the effectiveness of any therapy, including aptamers. Biological constraints emphasize the issue of insufficient knowledge about the implementation of aptamers; their design has evolved far faster than their incorporation into the complex process of GBM pathophysiology.

In addition to biological and technological factors, there is a systemic problem associated with the field: lack of standardization. Variation in SELEX protocols, antigen preparation techniques, buffer conditions, and analysis methodologies lead to inconsistencies and complicate data comparison. Inconsistencies result in difficulties with aptamer evaluation and identification of clinically applicable molecules. Standardization would help to accelerate research progress toward the clinical application of aptamers.

**Figure 6:**
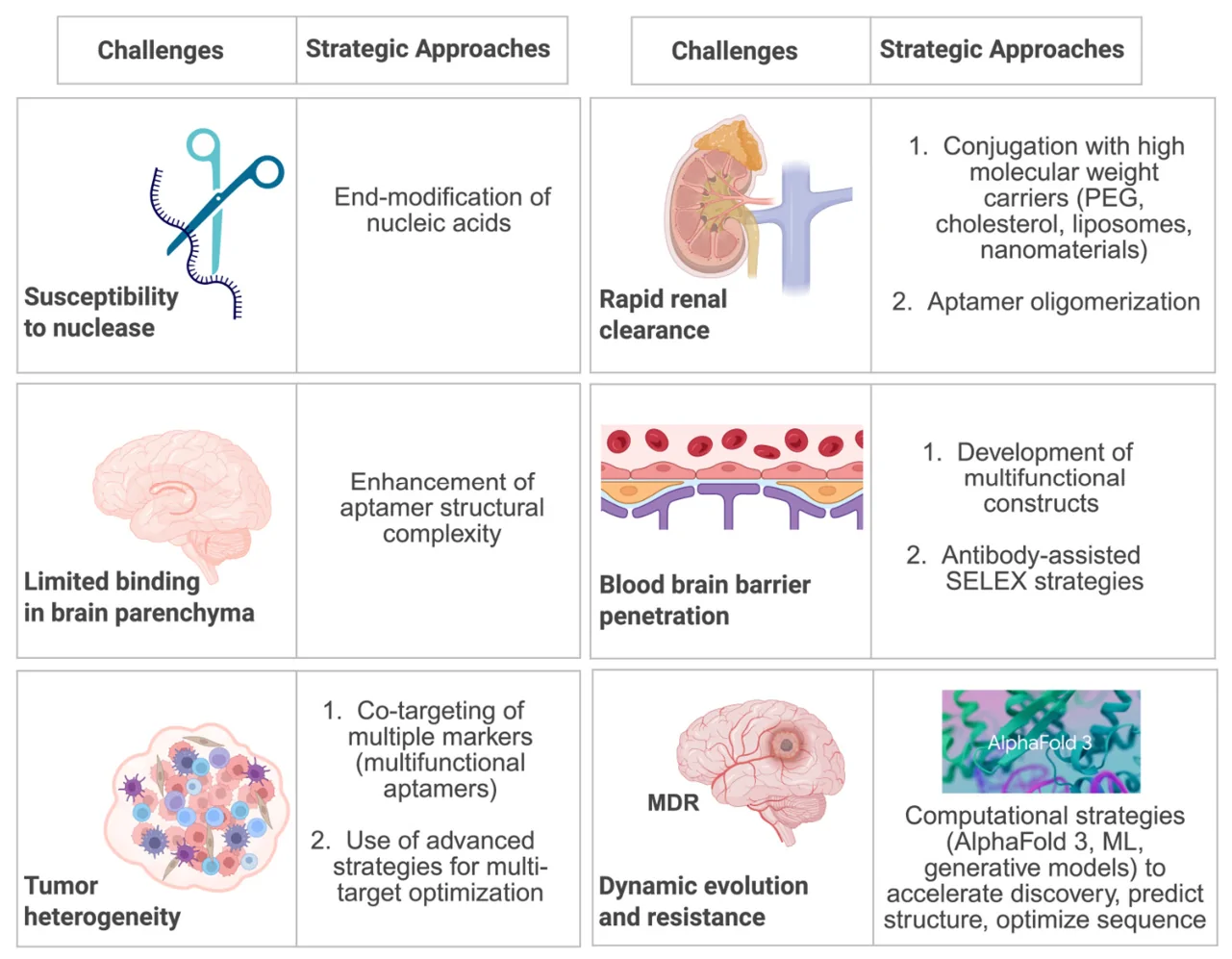
Overview of key challenges and corresponding strategic approaches in the development of aptamer-based therapeutics for brain targeting. Created in BioRender. Colasante, M. (2026) https://BioRender.com/t3rehd9 (accessed on 14 September 2026).

Methods such as structural prediction, machine learning-based optimization, generative modeling, and computational SELEX are providing novel ways forward, which are enabling the design of aptamers with enhanced capabilities to cross the BBB, improved resistance against nucleases, and multitarget recognition. Nonetheless, their full potential is hindered by the lack of quality training datasets as well as the intrinsic difficulties associated with aptamer–target binding studies under physiological conditions. As illustrated in Figure 6, the integration of aptamer engineering, nanotechnology and modeling of biosystems using artificial intelligence may one day enable the development of multi-functional theranostic platforms, which will be able to combine targeting, controlled release, and imaging. Nevertheless, discussing such an evolution remains speculative unless the critical translational challenges outlined above are overcome.

All of the above discussed factors are responsible for the creation of elegant molecular designs, which never go any further than promising preclinical studies. In order to move from theory to clinical application, it will not be enough to advance molecular engineering; parallel biological delivery systems, clinical trial design and regulatory frameworks will also be required.

## Figures and Tables

**Table 1:** Summary of Key Aptamers Developed for Glioblastoma (GBM) Therapy.

Aptamers	Target	Functional Role/Cargo	KD (nM)	Mechanism of Action	Developmental/Clinical Stage	References
AS1411	Nucleolin	Targeting Agent/Drug Delivery (e.g., Paclitaxel, Temozolomide)	69.1	Anti-proliferative, pro-apoptotic, and anti-migratory effects, with radio sensitizing activity in preclinical GBM models.	Phase II for other cancers (e.g., RCC, AML—discontinued); Preclinical for GBM.	[31]
U2	U87-EGFRvIII cells	Nanoparticles Delivery	6.27	Induction of apoptosis and inhibition of proliferation, cell migration and invasion	Preclinical (in vivo)	[32,33]
U8/U19/U31	EGFRvIII	Drug delivery	~2.5–8.1	Proliferation inhibition, radiosensitivity enhancement	Preclinical (in vitro)	[33]
CL4	EGFRvIII	Drug Delivery	10	EGFRvIII inhibition reducing migration, invasion and growth	Preclinical (in vivo)	[34]
Gint4.T	PDGFRβ	Drug Delivery (e.g., Paclitaxel, anti-miR-10b, siRNA)	9.6	Receptor-mediated uptake blocking growth signaling	Preclinical (in vivo)	[34,35]
A40s	EphA2 on GSCs	Therapeutics/ NATs Delivery	0.76 ± 0.2641	EphA2 binding inhibiting proliferation	Preclinical (in vivo)	[36,37,38]
GL43.T	EphB2/3 Receptors	Therapeutic Agent (Unconjugated)/Targeting Agent	433.5	Reduced cell vitality and EphB1-mediated adhesion inhibition	Preclinical (in vitro)	[39]
GBM128	Unknown	Targeting Agent	20 ± 10	Selective binding enabling receptor-mediated uptake	Preclinical (in vitro)	[40]
GBM131	Unknown	Targeting Agent	37 ± 13	Tumor-selective binding with internalization	Preclinical (in vitro)	[40]
A15	CD133	Targeting Agent/Drug Delivery/Theranostic	n.d.	CD133 targeting enabling BBB penetration and CSC targeting	Preclinical (in vitro)	[40]
E07 RNA aptamer	EGFR/EGFRvIII	Targeting Agent	2.4	Receptor autophosphorylation inhibition and GBM proliferation suppression	Preclinical (in vitro)	[41]
E21	EGFRvIII	Targeting Agent	33	Apoptosis induction	Preclinical (in vitro)	[42]
Aptamer 32 (A32)	EGFRvIII	Targeting Agent/Diagnostics/Drug Delivery (e.g., siRNA)	0.62 ± 0.04	EGFRvIII binding enabling targeted uptake	Preclinical (in vivo)	[43]
BA (5′-biotin-labeled Aptamer 32)	c-MET	Drug Delivery (e.g., siRNA)	n.d.	c-Met silencing, EGFRvIII-overexpressing glioma proliferation inhibition, and apoptosis induction	Preclinical (in vitro)	[44]
SL1	c-MET	Drug Delivery/Nanoparticles	123 ± 26	c-Met binding facilitating DOX delivery and apoptosis induction	Preclinical (in vitro and in vivo)	[45,46]
GBI-10	TNC (Tenascin-C)	Targeting Agent/Diagnostic	1.2	Tenascin-C binding	Preclinical (in vivo)	[47]
TTA1	TNC (Tenascin-C)	Targeting Agent	5	Tenascin-C binding	Preclinical (in vivo)	[48]
GL21.T	Axl Receptor Tyrosine Kinase	Targeting Agent/Drug Delivery (e.g., miR-137)	12	Axl targeting to inhibit invasion and drug resistance	Preclinical (in vitro and in vivo)	[49]
GMT8	Unknown	Targeting Agent/Theranostic/Nanoparticles		Inhibition of U87MG cell growth	Preclinical (in vitro and in vivo)	[50]
NOX-A12	CXCL12/SDF-1	Therapeutic Agent (Spiegelmer)	0.2	SDF-1 neutralization inhibiting signaling and disrupting chemokine-mediated tumor support	Phase 1/2 GLORIA TRIAL in newly diagnosed MGMT-unmethylated GBM; additional Phase II studies in other indications.	[51,52]
Pegaptanib	VEGF/VEGF165 isoform	Anti-angiogenic agent	0.05	Selective VEGF165 inhibition with anti-angiogenic activity in preclinical GBM models.	FDA approved for ophthalmologic use (neovascular AMD), with preclinical evaluation in GBM.	[53,54]
PDR3	PDGFRα	Targeting Agent	0.25	STAT3 expression suppression and JMJD3/p53 upregulation	Preclinical (in vitro)	[55]
CL-4RNV616	EGFR	Binding agent	18.24	U87MG cell proliferation inhibition	Preclinical (in vitro)	[56]
AS1411/GS24	Nucleolin/Transferrin Receptor	Drug delivery/Nanoparticle		Apoptosis and autophagy enhancement	Preclinical (in vitro and in vivo)	[57]
TfR Aptamers	Transferrin Receptor	Targeting Agent/Drug Delivery/Nanoplatform	<100	TfR binding enabling receptor-mediated uptake and targeted cargo delivery	Preclinical (in vitro and in vivo)	[58,59,60]
Hg1-9	Transferrin receptor	Targeting agent/Drug delivery	5.40 ± 0.721	Protein cargo transport across BBB	Preclinical (in vitro)	[58,60]
GL21.T/Gint4.T	AXL/PDGFRβ	Drug delivery/Nanoplatform		Apoptosis enhancement and inhibition of GBM cell migration, invasion, and proliferation	Preclinical (in vitro)	[61]
4-1BB–OPN bispecific aptamer (o 4-1BB/OPN)	4-1BB (CD137)/Osteopontin (OPN)	Bispecific targeting agent/Immunotherapy	2.32 (OPN/4-1BB)	Target-directed T-cell costimulation; recruits T cells to the OPN-rich tumor microenvironment	Preclinical (in vivo—GL261 immunocompetent mouse models)	[62]
GMT3	Unknown (A172 cell surface)	Drug delivery	75	Growth inhibition in GBM cell lines	Preclinical (in vitro)	[18]
TEPP	(EpCAM)/Transferrin Receptor	Targeting agent/Drug delivery	85.61 ± 28.49 (EpCAM) 110.3 ± 22.04 (TfR)	Selective binding to tumor endothelial progenitor cells inhibiting angiogenesis	Preclinical (in vitro and in vivo)	[63]
ATP DNA aptamer	ATP	DNA scaffold for DOX delivery	31 ± 3	Controlled and efficient DOX delivery system for glioma therapy	Preclinical (in vitro and in vivo)	[64,65]
W5-7	CD133 (putative)-GSCs	Targeting agent	4.9 ± 1.4	Not determined; no validated molecular target or functional mechanism reported	Preclinical (in vitro)	[66,67]
S6-1b	SHG44 cell-surface protein	Targeting agent	3.1 ± 0.6 nM at 4 °C and 44 ± 15 nM at 37 °C	Potential use for targeting, imaging, or delivery; no functional inhibition mechanism is reported.	Preclinical (in vitro)	[67]
hBS01–hBS06	Blood–Brain Barrier	Shuttle	2.8 (hBS01)	Protein cargo transport across BBB	Preclinical (in vitro and in vivo)	[68]
H02 RNA aptamer	A_5_β_1_^+^ integrin	Targeting agent	277.8 ± 51.8	Differentiation of glioblastoma cell lines and patient-derived xenograft tissues	Preclinical (in vitro)	[69]

**Table 2:** Comparative evaluation of chemical optimization strategies for therapeutic aptamers benchmarked against monoclonal antibodies (mAbs) and CAR-T cell therapies.

Therapeutic Platform/Chemistry	In Vivo Half-Life	Nuclease Stability	Immunogenicity	Cost & Chemical Scalability	References
2′-Fluoro (2′-F) Aptamers	Short to Moderate (Hours; prone to renal clearance)	High (Resistant to pyrimidine-specific nucleases)	Very Low/Negligible	Low to Moderate cost; High scalability (Automated solid-phase synthesis)	[15,81,82,83,84,94,95]
Locked Nucleic Acid (LNA) Aptamers	Moderate (Hours to Days)	Very High (Conformational locking protects sugar pucker)	Very Low	Moderate cost; High scalability (Standard chemical synthesis)	[15,84,96]
PEGylated Aptamers	Extended (Days; increased hydrodynamic radius mitigates renal filtering)	Moderate to High (Dependent on underlying sugar/backbone chemistry)	Low (Low incidence of anti-PEG antibodies reported)	Moderate cost; High scalability (Scalable chemical conjugation)	[84,97,98]
Spiegelmers (L-Nucleic Acid Aptamers)	Prolonged (Days; complete enzymatic resistance)	Exceptional/Total (Immune to natural nucleases due to inverted chirality)	Negligible (Non-immunogenic due to mirror-image stereochemistry)	Moderate to High cost; High scalability (Requires mirror-image D-target for selection)	[15,84,99]
Monoclonal Antibodies (mAbs)	Long (1–3 Weeks; protected by FcRn recycling)	N/A (Degraded by endopeptidases and proteolytic pathways)	Moderate to High (Risk of anti-drug antibodies [ADAs] and hypersensitivity)	High cost; Moderate scalability (Requires mammalian expression bioreactors)	[100]
CAR-T Cell Therapy	Variable to Long-Term (Weeks to Years; living therapeutic persistence)	N/A (Cleared via cellular apoptotic/immune clearance mechanisms)	High/Severe (Risk of Cytokine Release Syndrome [CRS] and ICANS)	Extremely High cost; Very Low scalability (Autologous cell engineering per patient)	[101,102]

Abbreviations: 2′-F, 2′-fluoro modification; LNA, locked nucleic acid; PEG, polyethylene glycol; mAbs, monoclonal antibodies; CAR-T, chimeric antigen receptor T-cell therapy; FcRn, neonatal Fc receptor; ADAs, anti-drug antibodies; CRS, cytokine release syndrome; ICANS, immune effector cell-associated neurotoxicity syndrome; N/A, not applicable.

**Table 3:** Nano-scaffold Platforms for Multivalent Aptamers Display.

Scaffold Type	Examples	Advantages	Limitations	Applications in GBM
DNA origami/RNA tiles	DNA origami sheets, RNA nanostructures	Programmable geometry; nanometer precision in aptamer spacing; high modularity	Complex synthesis; sensitive to nuclease degradation	Multivalent targeting of clustered receptors (EGFRvIII, PDGFRβ); logic-gated binding
Dendrimers	PAMAM dendrimers	High surface density of aptamers; tunable valency; good solubility	Potential cytotoxicity at high generations; batch variability	Enhanced avidity for heterogeneous receptor landscapes; drug delivery
Synthetic polymers	PEG, PLGA, polylysine scaffolds	Biocompatible; scalable; allow conjugation of drugs and aptamers	Less structural precision; possible steric hindrance	Co-delivery of aptamers with chemotherapeutics (Temozolomide, Doxorubicin)
Nanoparticles (inorganic/organic)	Gold NPs, silica NPs, liposomes	High stability; multifunctional cargo loading; imaging/radiosensitization	Risk of RES clearance; size-dependent BBB penetration	Radiosensitization (hafnium oxide NPs); aptamer-guided drug delivery
Protein scaffolds	Streptavidin tetramers, antibody fragments	Predictable valency; strong biotin–streptavidin interactions; modular assembly	Immunogenicity risk; limited flexibility	Bispecific aptamer display; immune modulation (e.g., 4-1BB co-stimulation)

Abbreviations: PAMAM, Poly (amidoamine); PEG, polyethylene glycol; PLGA, Poly(lactic-co-glycolic acid); NPs, nanoparticles.

## Data Availability

No new data was created or analyzed in this study. Data sharing is not applicable to this article.

## Supplementary Materials

The following supporting information can be downloaded at https://www.mdpi.com/article/10.3390/ijms27188231/s1.
