# Supplementary material for: Molecular Engineering of Aptamers for Glioblastoma Therapy: From Simple Antagonists to AI-Driven Approaches, a Narrative Review
Source: Int J Mol Sci. 2026 Sep 16;27(18):8231. doi: 10.3390/ijms27188231 (PMC13607356; doi:10.3390/ijms27188231)
Supplement: Supplementary file 1 [file ijms-27-08231-s001.zip › ijms-4444816-supplementary.pdf]

Review Methodology

A comprehensive literature search was conducted across electronic databases, including PubMed/MEDLINE, IEEE Xplore, Web of Science, and ClinicalTrials.gov, up to (January 2000 to June 2026). The search strategy utilized combinations of MeSH terms and relevant free-text keywords, including: *("aptamer" OR "nucleic acid aptamer" OR "Spiegelmer") AND ("glioblastoma" OR "GBM" OR "glioma" OR "brain tumor") AND ("target" OR "drug delivery" OR "clinical trial")*. Literature screening (title, abstract, and full text) was performed independently by two authors, with consensus reached for study inclusion. Reference lists of all eligible articles and previous reviews were manually screened to identify additional relevant publications.

Inclusion Criteria:

Original research articles, reviews, and clinical trial reports focusing on nucleic acid aptamers evaluated for glioblastoma therapy, diagnosis, or drug delivery. Studies reporting explicit molecular targets, binding affinity metrics (KD values), and characterized mechanisms of action (in vitro or in vivo). Early-phase clinical trials and translational studies evaluating aptamer safety, pharmacokinetics, or preliminary efficacy in GBM or solid tumors (e.g., NOX-A12, AS1411, Pegaptanib). Peer-reviewed studies published in English. Were only considered.

Exclusion Criteria:

Studies lacking specific data on aptamer sequences, binding affinities, or target characterization. Conference abstracts, editorials, commentaries, and non-peer-reviewed preprints without complete methodology. Non-English publications.

Data Extraction**:**

Data were extracted using a standardized template capturing aptamer nomenclature, chemical modifications, target specificity, KD values, biological mechanism of action, experimental model (*in vitro*, *in vivo*, or clinical trial stage), and translational status.

Supplementary Table S1: Comparative matrix of emerging algorithms, cancer complexity frameworks and translation-validation metrics.

Algorithm/ Method	Primary Translational Task	Typical Performance Metrics	Structural Limitations & Known Failure Modes	Benchmarking Status: Models Outperformed	Cancer Complexity Method & Translational Gap Addressed	Current Validation Status in GBM Systems	References
Generative AI models (AptaDiff)	*de novo* computational design and affinity-guided optimization of single-stranded DNA/RNA aptamers	FID Score: 1.06-2.51 (surpassing SOTA fidelity to real biological sequences). Structural Stability: Levenshtein distance and Minimum Free Energy (MFE) distributions match real HT-SELEX data. Affinity Boost: 87.9 increase in SPR RU values; up to 3.6-fold reduction in KD values.	Non-end-to-end training: Requires a separate, decoupled VAE pre-training phase to build the latent space.Lack of explicit 3D constraints: Sequence diffusion does not directly incorporate target pocket geometry or nucleic acid tertiary structures.	Outperforms RaptGen (SOTA VAE-HMM architecture) across all fidelity, GC-content, and structural metrics. Outperforms the top experimental hits selected via traditional, manual wet-lab HT-SELEX screening.	General-purpose Conditional Discrete Diffusion Model adapted for oncological targets via latent VAE feature extraction and Bayesian optimization	Early stage *in vitro* pre-clinical. Validated strictly via Surface Plasmon Resonance (SPR) using isolated, purified target proteins. No validation for GBM.	[186]
in Silico SELEX (RaptScore)	Computational selection and 3D optimization of tumor-targeting RNA/DNA aptamers.	Spearman 0.65-0.74, Pearson 0.62-0.69) for binding activity ranking. In silico maturation, a 10-nucleotide	Lack of 3D atomic coordinates, reliance on sequence-level context and susceptibility to dataset specific	Outperforms traditional frequency-based counts, standard masked language models, DeepLnc classifiers, and	Evaluated on general RNA protein binding datasets. Bypasses PCR bias shortens sequences and finds high affinity candidates.	No specific GBM or oncological *in vitro/in vivo* validation	[191]

		sequence truncation with 100% efficiency and a 2.5-fold to 3.6-fold increase in high affinity candidate discovery using Raptgen.	biases. *In vitro* inactive sequeces or false positive.	classical generative frameworks in binding activity prediction.			
Prediction framework (AlphaFold3)	All-atom biomolecular structure prediction (proteins, ligands, nucleic acids, ions)	Pocket-aligned ligand RMSD < 2 Å (PoseBusters benchmark) TM-score (global accuracy) LDDT (local accuracy) DockQ (protein–protein interfaces) FNAT (protein–nucleic acid interfaces) INF (RNA interaction fidelity) CDR-TMscore (antibody loops)	Chirality violations (~4.4%); occasional atom clashes in large protein–nucleic complexes; hallucinated structural order in disordered regions; variability in ligand placement, especially in cryptic or partially formed pockets	Outperforms AlphaFold2 (multimer), RoseTTAFold All-Atom, and classical AutoDock Vina	Not specifically designed to address cancer complexity; AF3 operates as a structural prediction engine without dedicated modules for tumor heterogeneity, microenvironmental dynamics, or translational modeling. Validated only on structural benchmarks and not on pathological or oncogenic systems	No experimental validation in GBM models	[178,192,193]

POTFlow Framework	Lead-peptide-driven generative optimization of therapeutic binders.	Structural Similarity: 53.44%; Compactness: 95.07%; Binding Affinity: 30.56%; Stabiliy: 23.80; RMSD: 1.66, BSR: 87.01.	Lead-dependent optimization, limited sequence-space exploration, secondary-structure dependency, bias toward known motifs, OT mapping sensitivity, reduced conformational flexibility, training-data dependence, limited target generalizability, computational experimental gap, and potential loss of diversity.	Demonstrates statistical improvements over *RFDiffusion*, *ProteinGenerator*, *PepFlow*, *PepGLAD*, and *PepHAR*.	Geometry-Aware Multimodal Flow-Matching: Couples class-specific structure priors (DSSP) with intra-sample Optimal Transport (OT).	Dry-to-Wet Validation: Evaluated via selective ATP depletion/cytotoxicity *in vitro*; tumor suppression and survival benefit shown in orthotopic PDX *in vivo*.	[198]
